# Effect of Levocarnitine vs Placebo as an Adjunctive Treatment for Septic Shock: The Rapid Administration of Carnitine in Sepsis (RACE) Randomized Clinical Trial

**DOI:** 10.1001/jamanetworkopen.2018.6076

**Published:** 2018-12-21

**Authors:** Alan E. Jones, Michael A. Puskarich, Nathan I. Shapiro, Faheem W. Guirgis, Michael Runyon, Jason Y. Adams, Robert Sherwin, Ryan Arnold, Brian W. Roberts, Michael C. Kurz, Henry E. Wang, Jeffrey A. Kline, D. Mark Courtney, Stephen Trzeciak, Sarah A. Sterling, Utsav Nandi, Deepti Patki, Kert Viele

**Affiliations:** 1 Department of Emergency Medicine, The University of Mississippi Medical Center, Jackson; 2 Department of Emergency Medicine, Hennepin County Medical Center, Minneapolis, Minnesota; 3 Department of Emergency Medicine, Beth Israel Deaconess Medical Center, Boston, Massachusetts; 4 Department of Emergency Medicine, University of Florida College of Medicine–Jacksonville; 5 Department of Emergency Medicine, Carolinas Medical Center, Charlotte, North Carolina; 6 Division of Pulmonary, Critical Care, and Sleep Medicine, Department of Internal Medicine, University of California, Davis; 7 Department of Emergency Medicine, Wayne State University, Detroit, Michigan; 8 Department of Emergency Medicine, Christiana Care Health System, Wilmington, Delaware; 9 Department of Emergency Medicine, Cooper University Hospital, Cooper Medical School of Rowan University, Camden, New Jersey; 10 Department of Emergency Medicine, The University of Alabama School of Medicine at Birmingham; 11 Department of Emergency Medicine, The University of Texas Health Science Center at Houston; 12 Department of Emergency Medicine, Indiana University School of Medicine, Indianapolis; 13 Department of Emergency Medicine, Northwestern University, Chicago, Illinois; 14 Department of Medicine, Cooper University Hospital, Cooper Medical School of Rowan University, Camden, New Jersey; 15 Berry Consultants, Austin, Texas

## Abstract

**Importance:**

Sepsis induces profound metabolic derangements, while exogenous levocarnitine mitigates metabolic dysfunction by enhancing glucose and lactate oxidation and increasing fatty acid shuttling. Previous trials in sepsis suggest beneficial effects of levocarnitine on patient-centered outcomes.

**Objectives:**

To test the hypothesis that levocarnitine reduces cumulative organ failure in patients with septic shock at 48 hours and, if present, to estimate the probability that the most efficacious dose will decrease 28-day mortality in a pivotal phase 3 clinical trial.

**Design, Setting, and Participants:**

Multicenter adaptive, randomized, blinded, dose-finding, phase 2 clinical trial (Rapid Administration of Carnitine in Sepsis [RACE]). The setting was 16 urban US medical centers. Participants were patients aged 18 years or older admitted from March 5, 2013, to February 5, 2018, with septic shock and moderate organ dysfunction.

**Interventions:**

Within 24 hours of identification, patients were assigned to 1 of the following 4 treatments: low (6 g), medium (12 g), or high (18 g) doses of levocarnitine or an equivalent volume of saline placebo administered as a 12-hour infusion.

**Main Outcomes and Measures:**

The primary outcome required, first, a greater than 90% posterior probability that the most promising levocarnitine dose decreases the Sequential Organ Failure Assessment (SOFA) score at 48 hours and, second (given having met the first condition), at least a 30% predictive probability of success in reducing 28-day mortality in a subsequent traditional superiority trial to test efficacy.

**Results:**

Of the 250 enrolled participants (mean [SD] age, 61.7 [14.8] years; 56.8% male), 35, 34, and 106 patients were adaptively randomized to the low, medium, and high levocarnitine doses, respectively, while 75 patients were randomized to placebo. In the intent-to-treat analysis, the fitted mean (SD) changes in the SOFA score for the low, medium, and high levocarnitine groups were −1.27 (0.49), −1.66 (0.38), and −1.97 (0.32), respectively, vs −1.63 (0.35) in the placebo group. The posterior probability that the 18-g dose is superior to placebo was 0.78, which did not meet the a priori threshold of 0.90. Mortality at 28 days was 45.9% (34 of 74) in the placebo group compared with 43.3% (45 of 104) for the most promising levocarnitine dose (18 g). Similar findings were noted in the per-protocol analysis.

**Conclusions and Relevance:**

In this dose-finding, phase 2 adaptive randomized trial, the most efficacious dose of levocarnitine (18 g) did not meaningfully reduce cumulative organ failure at 48 hours.

**Trial Registration:**

ClinicalTrials.gov Identifier: NCT01665092

## Key Points

**Question:**

Do 1 or more doses of levocarnitine reduce organ failure in septic shock at 48 hours, and, if so, what is the likelihood of success in a phase 3 trial?

**Findings:**

In an adaptive randomized, blinded clinical trial of 250 adults, the most efficacious dose of levocarnitine (18 g) demonstrated a posterior probability of efficacy of 0.78, which did not reach the a priori threshold of 0.90.

**Meaning:**

Levocarnitine did not meaningfully reduce organ failure at 48 hours in patients with septic shock.

## Teaser

This randomized clinical trial tests the hypothesis that levocarnitine reduces cumulative organ failure in patients with septic shock at 48 hours and estimates the probability that the most efficacious dose will decrease 28-day mortality.

## Introduction

Sepsis is the leading cause of death in the intensive care unit (ICU),^1^ and mortality approaches 40% at 28 days when shock is present.^2^ Sepsis accounts for $24 billion of annual inpatient hospital costs, making it the most expensive condition to treat in the United States.^3^ Initial care for sepsis consists of early recognition and stabilization, combined with adequate source control through the use of broad-spectrum antibiotics and/or surgical intervention.^4^ Current standard practice to support failing organs is generally limited to fluid resuscitation, vasopressors, ventilatory support, and renal replacement therapy, as necessary. Despite significant research investment over the last 5 decades, there remains a lack of specific pharmacologic therapies targeting the pathophysiology of sepsis. Given the high mortality rate and limited therapeutic options, development of novel treatments remains a priority.

Metabolic abnormalities in sepsis include hyperglycemia, hyperlactatemia, ketosis, and increased free fatty acids. Vital organs, such as the heart, change fuel preference in response to a septic insult.^5^ The degree of metabolic disturbance, characterized by the severity of hyperlactatemia, is strongly predictive of death.^6,7^ While often considered an indicator of tissue hypoperfusion and anaerobic metabolism, increased lactate production can result from pyruvate dehydrogenase complex inhibition in sepsis.^8^ Levocarnitine may mitigate some of the metabolic effects of sepsis by simultaneously enhancing fatty acid entry into the mitochondria, clearing their toxic effects from the cytosol and sequestering intramitochondrial acetate, leading to a decrease in the inhibitory effect of acetyl–coenzyme A on the pyruvate dehydrogenase complex.^9,10^ Preclinical animal models^11,12^ and 2 small clinical trials of acetyl-l-carnitine^13^ and l-carnitine^14^ have demonstrated their relative safety and potential efficacy in the reduction of organ dysfunction and possibly mortality, although the clinical trials were limited by small numbers of participants and different outcome reporting.

To better understand the potential effects of levocarnitine as a metabolic therapy for sepsis, we conducted a multicenter adaptive, randomized, blinded, dose-finding, phase 2 clinical trial to compare 3 doses of levocarnitine vs placebo. The hypotheses of this study were that 1 or more doses of levocarnitine would reduce cumulative organ failure compared with placebo and that the most promising dose of levocarnitine would have at least a 30% probability of demonstrating efficacy in the reduction of 28-day mortality if carried forward into a pivotal phase 3 clinical trial.

## Methods

### Study Design

This study was a prospective, randomized, blinded, placebo-controlled clinical trial using Bayesian response-adaptive randomization and designed to assess the efficacy of levocarnitine to decrease the Sequential Organ Failure Assessment (SOFA) score (originally the Sepsis-Related Organ Failure Assessment)^15^ of patients with septic shock. The SOFA score (range, 0-24) is an organ failure scoring system consisting of 6 physiological systems; a score of 2 or higher is required for the diagnosis of sepsis. The trial took place from March 5, 2013, to February 5, 2018, in the emergency departments and ICUs of 16 large urban medical centers in the United States. The research protocol was approved by the local institutional review boards and was performed in accord with Good Clinical Practice guidelines. This study followed the Consolidated Standards of Reporting Trials (CONSORT) reporting guideline.^16^ The trial protocol is available in Supplement 1.

### Participants

Patients seen at the participating centers during the study period with septic shock and moderate organ dysfunction were assessed for inclusion. Criteria for inclusion were as follows: (1) patients 18 years or older with confirmed or presumed infection; (2) the presence of 2 or more systemic inflammatory response criteria; (3) enrollment within 24 hours of recognition of septic shock with initiation of a standardized sepsis treatment pathway; (4) the use of high-dose vasopressors (norepinephrine bitartrate >0.05 μg/kg/min, dopamine hydrochloride >10 μg/kg/min, phenylephrine hydrochloride >0.4 μg/kg/min, epinephrine >0.05 μg/kg/min, or any vasopressin dose) to treat shock for at least 4 hours at the time of enrollment; (5) cumulative SOFA score of at least 6; (6) and blood lactate level exceeding 18 mg/dL (to convert lactate level to millimoles per liter, multiply by 0.111). Patients were excluded if they were pregnant or breastfeeding or had any of the following characteristics: primary diagnosis other than sepsis, an established do-not-resuscitate status or advance directive restricting aggressive care, any history of seizures, known inborn error of metabolism, anticipated surgery that would interfere with a 12-hour infusion, active participation in another interventional trial, cardiopulmonary resuscitation before enrollment, known allergy to levocarnitine, active warfarin treatment, or severe immunocompromised state (absolute neutrophil count, <500/μL; to convert neutrophil count to ×10^9^/L, multiply by 0.001).

Eligible patients were screened 7 days per week using electronic medical record–based reports of admitted patients receiving both antibiotics and vasopressors and/or pager-based alerts informing study staff regarding initiation of a sepsis treatment pathway (which composed the screening log), in addition to active screening of the emergency department tracking board to facilitate earlier identification. Each enrolled patient or the patient’s legally authorized representative provided written informed consent before randomization and collection of data.

### Treatment Assignment

After informed consent was obtained, a centralized web-based portal was used to determine treatment allocation. Patients were randomly assigned to 1 of 3 doses of levocarnitine or saline placebo. During an initial “burn-in” period of 40 patients, participants were allocated equally among the treatment arms. From that point on, interim analyses were conducted on every 12 patients. At each interim analysis, the relative allocation probability of the 3 active treatment arms was adjusted to be proportional to the probability that each arm would lead to the greatest improvement in the SOFA score. A site-based blocked randomization approach ensured that approximately one-third of participants were allocated to the control arm throughout the trial to maintain a sufficient allocation to control and avoid confounding from local changes in usual care. Predefined stopping rules for both efficacy and futility were determined before trial initiation. The statistical rationale and methods have been published previously.^17^

### Treatment Interventions

The 3 active treatment arms consisted of low (6 g), medium (12 g), and high (18 g) doses of levocarnitine administered intravenously over a 12-hour period. The control (placebo) consisted of an identical volume of 0.9% saline placebo. After randomization, research pharmacists used a web-based portal to determine treatment allocation. Pharmacists were the only individuals not masked to treatment, and they had no study-related contact with the investigators or participants. Pharmacists and staff prepared either levocarnitine or placebo in identical polypropylene infusion bags with labels that included the study identification number, patient name, medical record number, and infusion rate. For each dose of levocarnitine, 33% of the total dose was administered as a 20-mL bolus over 2 to 3 minutes, followed by a fixed-rate continuous infusion of 1 L over the next 12 hours. The study solution was administered through intravenous (IV) catheters using US Food and Drug Administration–approved medical equipment (IV tubing, IV pumps, etc). Levocarnitine was provided by Leadiant Biosciences (formerly Sigma-Tau Pharmaceuticals) and maintained by pharmacy staff, with tracking of lot numbers of levocarnitine administered. For safety, clinical physicians could elect to break study masking, although in no instance did this occur.

### Assessments and Outcome Measures

During the study treatment period, the patient’s physiological variables, laboratory results, and medical treatments were recorded. Sex and race/ethnicity were self-reported. An investigator performed a bedside assessment and recorded vital signs, vasopressor requirements, ventilator settings, and Glasgow Coma Scale score at enrollment and 12, 24, and 48 hours later. At enrollment and 48 hours, blood samples were sent to the clinical laboratory for platelet count, creatinine level, and total bilirubin level to ensure capture of the SOFA score variables. Nonprotocol (clinical) laboratory results obtained between 0 and 48 hours were recorded. Patients were followed up until hospital discharge or death and then up to 1 year using the patient’s electronic medical record and phone calls to the patient or legally authorized representative, cross-referenced with the Social Security Death Index. Study data were collected and managed using research electronic data capture (REDCap).^18^

The primary end points were change in the SOFA scores from enrollment to 48 hours, with negative numbers indicating improvement, and 28-day mortality. In the event of early death before 48 hours, last value carried forward was used. Secondary outcomes included ICU and hospital length of stay and percentage of patients undergoing withdrawal of care. Three preplanned, blinded interim safety analyses were performed: first, after a burn-in phase of 40 patients and then after one-third and two-thirds of participants were enrolled. The unblinded results were reviewed by an independent data safety monitoring board with the authority to terminate the study for safety concerns or if the predefined stopping rules for efficacy or futility were fulfilled.

### Statistical Analysis

The trial was considered to have positive findings if (1) the posterior probability of any dose decreasing the SOFA score at 48 hours more than placebo exceeded 90% and (2) (having met the first condition) there was at least a 30% predictive probability that the most promising dose of levocarnitine would be successful in reducing 28-day mortality in a subsequent 2-arm, 2000–total patient phase 3 trial. Based on Monte Carlo simulation of 30 000 simulated trials enrolling up to 250 patients, the probability of a positive trial assuming no treatment effect (the type I error rate α) was 4.3%. The power of the trial (β) was dependent on the true treatment effect. If the true SOFA score effects for the 3 levocarnitine treatment arms were 0, 1, and 2, corresponding to mortality effects of 0%, 6%, and 12%, respectively, then the power of the trial was 91.1%.

Changes in the SOFA scores between groups were analyzed using a Bayesian approach assuming a normal dynamic linear model dose response, and posterior probabilities are reported. The normal dynamic linear model is a Bayesian analogue to a smoothing spline, whose smoothness is determining by a tuning variable. The tuning variable is given a priori and thus is determined by a combination of the prior and the observed data. The prior was selected during the design process to provide a smooth fit, while maintaining the main features of the observed data.

Conditional on a declaration of the SOFA score reduction, predictive probabilities of success in a subsequent phase 3 trial were calculated using noninformative priors. Except for these primary outcomes, remaining data were analyzed using frequentist statistics. Categorical data were compared using χ^2^ tests, while continuous variables were compared with analysis of variance or Wilcoxon rank sum test based on normality. All non-Bayesian tests were 2-sided, with *P* < .05 considered significant. All trial design simulations, interim analyses, and Bayesian statistics were performed using the Fixed and Adaptive Clinical Trial Simulator Software (FACTS) (Berry Consultants, LLC), while frequentist statistics were conducted using Stata 15.1 (StataCorp).

## Results

**Figure 1.**
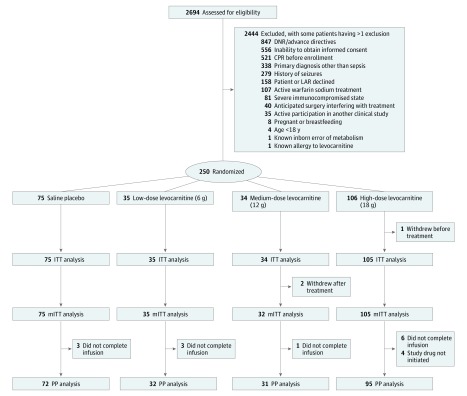
Consolidated Standards of Reporting Trials (CONSORT) Study Flow Diagram CPR indicates cardiopulmonary resuscitation; DNR, do not resuscitate; ITT, intent-to-treat; LAR, legally authorized representative; mITT, modified intent-to-treat; and PP, per-protocol.

We screened 2694 individuals and enrolled 250 patients (mean [SD] age, 61.7 [14.8] years; 56.8% male). The most efficacious dose of levocarnitine (18 g) demonstrated 78% and 77% probabilities of superiority compared with placebo in the intent-to-treat (ITT) and per-protocol (PP) analyses, respectively. The CONSORT study flow diagram and reasons for exclusion are shown in Figure 1. Groups were well matched for baseline demographics, comorbidities, physiological variables, and severity of illness measurements with the exception of age, which was older in the low- and medium-dose groups, and Acute Physiology and Chronic Health Evaluation (APACHE) II scores,^19^ which were lowest in the high-dose group and highest in the medium-dose group despite well-matched SOFA scores (Table 1). Interventions administered did not differ significantly between groups (Table 2).

**Table 1.** Baseline Demographics and Clinical Characteristics of Patients by Treatment Allocation

Variable	Saline Placebo (n = 75)	Low-Dose Levocarnitin, 6 g (n = 35)	Medium-Dose Levocarnitine, 12 g (n = 34)	High-Dose Levocarnitine, 18 g (n = 106)
Age, mean (SD), y	60.22 (16.50)	67.51 (12.01)	64.91 (12.56)	59.67 (14.50)
Race, No. (%)				
White	47 (62.7)	25 (71.4)	21 (61.8)	55 (51.9)
Black	25 (33.3)	8 (22.9)	8 (23.5)	39 (36.8)
Asian	0	1 (2.9)	1 (2.9)	3 (2.8)
Other	3 (4.0)	1 (2.9)	4 (11.8)	9 (8.5)
Ethnicity, No. (%)				
Hispanic	3 (4.0)	0	3 (8.8)	6 (5.7)
Non-Hispanic	72 (96.0)	35 (100)	31 (91.2)	99 (93.4)
Sex, No. (%)				
Male	39 (52.0)	18 (51.4)	21 (61.8)	64 (60.4)
Female	36 (48.0)	17 (48.6)	13 (38.2)	42 (39.6)
Comorbidities, No. (%)				
Diabetes	26 (34.7)	15 (42.9)	7 (20.6)	37 (34.9)
Chronic obstructive pulmonary disease	16 (21.3)	7 (20.0)	5 (14.7)	20 (18.9)
Congestive heart failure	14 (18.7)	3 (8.6)	9 (26.5)	21 (19.8)
Cerebrovascular accident	7 (9.3)	5 (14.3)	2 (5.9)	15 (14.2)
HIV	1 (1.3)	0	0	5 (4.7)
End-stage renal disease	5 (6.7)	3 (8.6)	3 (8.8)	11 (10.4)
Active cancer	12 (16.0)	8 (22.9)	3 (8.8)	12 (11.3)
Organ transplant	0	1 (2.9)	2 (5.9)	1 (0.9)
Indwelling vascular line	9 (12.0)	5 (14.3)	6 (17.6)	17 (16.0)
Nursing home resident	9 (12.0)	4 (11.4)	5 (14.7)	16 (15.1)
Disease severity, median (IQR)				
Enrollment SOFA score	12 (8-14)	11 (9-15)	12 (8-14)	11 (9-13)
Enrollment APACHE II score	23 (16-28)	25 (17-29)	24 (18-30)	19 (14-25)
Enrollment lactate level, mg/dL	32.4 (23.4-58.6)	46.8 (25.2-66.7)	30.6 (18.9-68.5)	30.6 (20.7-54.1)
Suspected source of infection, No. (%)				
Pulmonary	27 (36.0)	12 (34.3)	12 (35.3)	30 (28.3)
Urinary tract	13 (17.3)	6 (17.1)	7 (20.6)	15 (14.2)
Intra-abdominal	8 (10.7)	6 (17.1)	2 (5.9)	13 (12.3)
Skin/soft tissue	4 (5.3)	1 (2.9)	1 (2.9)	10 (9.4)
Other	16 (21.3)	5 (14.3)	9 (26.5)	23 (21.7)
Unknown	7 (9.3)	5 (14.3)	3 (8.8)	15 (14.2)
Features of sepsis, No./total No. (%)				
Culture positive	23/75 (30.7)	10/35 (28.6)	9/34 (26.5)	37/105 (35.2)
Culture negative	52/75 (69.3)	25/35 (71.4)	25/34 (73.5)	68/105 (64.8)

Abbreviations: APACHE, Acute Physiology and Chronic Health Evaluation (score range, 0-71, with higher values indicating greater organ failure); IQR, interquartile range; SOFA, Sequential Organ Failure Assessment (score range, 0-24, with higher values indicating greater organ failure).

SI conversion factor: To convert lactate level to millimoles per liter, multiply by 0.111.

**Table 2.** Clinical Interventions and Administered Therapies by Treatment Allocation

Variable	Saline Placebo (n = 75)	Low-Dose Levocarnitine, 6 g (n = 35)	Medium-Dose Levocarnitine, 12 g (n = 34)	High-Dose Levocarnitine, 18 g (n = 106)	*P* Value
Fluids administered, mean (SD), L					
24 h Before enrollment	3.80 (2.11)	4.02 (2.92)	4.40 (3.08)	3.94 (2.66)	.77
6 h After initiation of hemodynamic resuscitation	3.39 (6.00)	3.12 (1.86)	2.74 (1.90)	2.52 (1.89)	.50
Mechanical ventilation					
No. (%)	55 (73.3)	29 (82.9)	23 (67.6)	78 (73.6)	.54
Total time, median (IQR), h	113.1 (64.0-252.6)	127.8 (53.7-210.3)	161.5 (46.0-306.7)	98.6 (41.7-191.8)	.46
Corticosteroids, No. (%)	28 (37.3)	14 (40.0)	17 (50.0)	40 (37.7)	.64
Hemodialysis, No. (%)	16 (21.3)	4 (11.4)	2 (5.9)	16 (15.1)	.18

Abbreviation: IQR, interquartile range.

One patient in the high-dose group requested withdrawal before initiation of infusion, leaving 249 patients for the ITT analysis. After the 40-patient burn-in phase of equal allocation across all 4 treatment arms, 45.7% (96 of 210) of patients were preferentially randomized to the high dose while maintaining approximately one-third (31.0% [65 of 210]) in the placebo arm. In total, 35, 34, and 106 patients were adaptively randomized to the low, medium, and high levocarnitine doses, respectively, with 75 patients randomized to placebo. Forty-one patients died before the 48-hour time point and required last SOFA score carried forward to calculate the primary outcome. Two additional patients withdrew after treatment, permitting no 28-day mortality assessment, leaving 247 patients for the modified ITT analysis. An additional 17 patients never began (n = 4) or did not complete (n = 13) the 12-hour infusion. After exclusion of these patients and withdrawals, 230 patients remained for the PP analysis. Reasons for failing to begin the infusion included actual or imminent death after consent but before drug infusion (n = 3) and insufficient IV access to allow study drug infusion (n = 1). Reasons for early cessation of infusion included death before 12 hours (n = 7), family request to stop infusion (n = 2), withdrawal of care or transition to comfort measures (n = 2), local allergic reaction to infusion (n = 1), and patient request due to development of burning sensation in the throat (n = 1).

**Figure 2.**
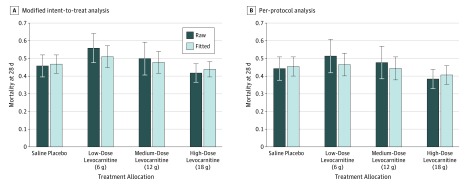
Raw and Fitted Mortality Rates of Patients Treated With Saline Placebo or 6, 12, or 18 g of Levocarnitine Based on the Modified Intent-to-Treat and Per-Protocol Analyses Error bars represent SDs around raw and model-fitted mortality rates.

The mean (SD) baseline SOFA score was 11.1 (3.6), and the overall 28-day mortality rate was 46.6% (115 of 247). The raw and fitted change in the SOFA scores and 28-day mortality rates for the ITT and PP analyses, as well as the probability that each dose is more efficacious than placebo, are listed in Table 3. In the ITT analysis, the fitted mean (SD) changes in the SOFA score for the low, medium, and high levocarnitine groups were −1.27 (0.49), −1.66 (0.38), and −1.97 (0.32), respectively, vs −1.63 (0.35) in the placebo group. The posterior probability that the 18-g dose is superior to placebo was 0.78, which did not meet the a priori threshold of 0.90. Mortality at 28 days was 45.9% (34 of 74) in the placebo group compared with 43.3% (45 of 104) for the most promising levocarnitine dose (18 g). The raw and fitted mortality rates for both the modified ITT and PP analyses are shown in Figure 2. The high (18 g) dose of levocarnitine demonstrated the most favorable raw (0.35 in ITT) and fitted (0.34 in ITT) decreases in the SOFA score for the primary outcome at 48 hours in both the ITT and PP analyses. None of these changes met the a priori threshold for study success. The high dose showed the most efficacious effect on 28-day mortality, with a modest (3%) decrease in the modified ITT analysis but a more clinically significant effect (5%-6% decrease) in the PP analyses. The predicted probabilities of success of high-dose carnitine in a phase 3 pivotal trial with a mortality end point in the ITT and PP analyses were 0.40 and 0.50, respectively.

**Table 3.** Raw and Fitted Change in the SOFA Scores (48-Hour SOFA Score Minus Enrollment SOFA Score) for the Intent-to-Treat and Per-Protocol Analyses^a^

Variable	Raw Mean (SE)	Raw Treatment Effect	Fitted Mean (SD)	Fitted Treatment Effect (SD)	Posterior Probability of Treatment Superior to Placebo	Mortality at 28 d, No./Total No. (%)	Model Fitted Rate (SD)	Predictive Probability of Phase 3 Success
**Intent-to-Treat Analysis (n = 249)**								
Saline placebo (n = 75)	−1.80 (0.35)	NA	−1.63 (0.35)	NA	NA	34/74 (45.9)	0.47 (0.05)	NA
Low-dose levocarnitine, 6 g (n = 35)	−0.34 (0.66)	−1.46	−1.27 (0.49)	−0.37 (0.52)	0.24	20/34 (58.8)	0.51 (0.06)	0.10
Medium-dose levocarnitine, 12 g (n = 34)	−1.68 (0.61)	−0.12	−1.66 (0.38)	0.03 (0.47)	0.54	16/32 (50.0)	0.48 (0.06)	0.22
High-dose levocarnitine, 18 g (n = 105)	−2.15 (0.37)	0.35	−1.97 (0.32)	0.34 (0.44)	0.78	45/104 (43.3)	0.44 (0.04)	0.40
**Per-Protocol Analysis (n = 230)**								
Saline placebo (n = 72)	−1.91 (0.38)	NA	−1.78 (0.36)	NA	NA	32/71 (45.1)	0.46 (0.05)	NA
Low-dose levocarnitine, 6 g (n = 32)	−0.57 (0.75)	−1.34	−1.53 (0.48)	−0.25 (0.48)	0.32	16/31 (51.6)	0.47 (0.06)	0.17
Medium-dose levocarnitine, 12 g (n = 31)	−1.94 (0.65)	0.03	−1.87 (0.38)	−0.09 (0.46)	0.59	15/31 (48.4)	0.45 (0.06)	0.28
High-dose levocarnitine, 18 g (n = 95)	−2.31 (0.40)	0.40	−2.12 (0.33)	0.33 (0.45)	0.77	37/94 (39.4)	0.41 (0.05)	0.50

Abbreviations: NA, not applicable; SOFA, Sequential Organ Failure Assessment (score range, 0-24, with higher values indicating greater organ failure).

^a^ The predictive probability of phase 3 success has been computed with noninformative priors. In the absence of meeting the required success threshold for the SOFA score change, these predictive probabilities should be interpreted skeptically. Negative raw mean and positive treatment effects on the SOFA scores reflect greater reductions in the SOFA scores and indicate clinical improvement.

We observed no significant differences in the median ICU length of stay across the placebo (7 days; interquartile range [IQR], 3-15 days), low-dose (6 days; IQR, 4-11 days), medium-dose (7 days; IQR, 3-13 days), and high-dose (6 days; IQR, 3-15 days) arms (*P* = .90). Additional median numbers of hospital days after ICU discharge did not differ significantly: 8 days (IQR, 1-18 days), 4 days (IQR, 1-13 days), 5 days (IQR, 1-17 days), and 6 days (IQR, 0-14 days), respectively (*P* = .48). We observed no difference in the proportion of patients who had care withdrawn: 0.13, 0.20, 0.12, and 0.07, respectively (*P* = .15). The median number of ICU days was 8 days (IQR, 4-20 days) for survivors vs 5 days (IQR, 2-10 days) for nonsurvivors. The median number of non-ICU hospital days was 16 days (IQR, 9-28 days) among survivors vs 7 days (IQR 3-14 days) among nonsurvivors.

## Discussion

In this adaptive, double-blinded, parallel-group randomized clinical trial, we randomized 250 patients with septic shock and moderate organ dysfunction to treatment with 1 of 3 doses of levocarnitine or to saline placebo. The most efficacious dose of levocarnitine (18 g) demonstrated 78% and 77% probabilities of superiority compared with placebo in the ITT and PP analyses, respectively. For study interpretation, standard 95% CIs can be calculated using the raw mean (SE) of ±1.96 listed in Table 3. Therefore, the study did not meet the a priori 90% posterior probability of a reduction in the SOFA score required.

As opposed to the majority of sepsis trials investigating ancillary pharmacologic agents that have focused on modulation of inflammation^20,21^ or coagulation,^22,23,24,25^ this approach to sepsis pharmacotherapy was innovative in that we attempted to target one of the known metabolic derangements of sepsis. Sepsis-induced alterations of metabolism are diverse and predictive of outcome.^26^ Most trials in sepsis target inflammatory or coagulation cascades, while examples of metabolic approaches to the condition are sparse. Although insulin therapy has been extensively investigated in critically ill patients, including those with sepsis with mixed results,^27,28,29^ most studies have targeted glycemic control. Trials of micronutrients and feeding regimens, often with the implicit aim of correcting deficiencies, have been investigated, but strong evidence for specific micronutrient provision beyond early enteral feeding remains lacking.^30^ In this trial, we sought to supplement all patients with a metabolic nutrient, with the goal of improving organ function regardless of underlying deficiency. Although our trial did not meet a priori efficacy outcomes, metabolic manipulation may still represent a viable therapeutic target with alternative metabolically active agents.

We conducted this trial in undifferentiated patients with septic shock and moderate organ dysfunction, but it has become increasingly apparent that sepsis represents a heterogeneous condition, which may contribute to the poor historical success rates. Future phenotyping through the use of pharmacometabolomics^31^ may identify patients amenable to metabolic support and increase the likelihood of trial success. Phenotyping a cohort of patients enrolled in a smaller, similar trial of levocarnitine demonstrated a clinically occult cohort who appeared more responsive to treatment and showed disproportionate clinical benefit.^32^ It remains possible that levocarnitine drug responsiveness may be predictable based on metabolic phenotyping or assessment of levocarnitine deficiency and provide more precise enrollment criteria for subsequent studies.

Independent of the efficacy of levocarnitine, this trial demonstrates the viability of a Bayesian adaptive randomization design in acute critical illness trials. The adaptive randomization design preferentially allocated patients toward the most efficacious high dose and away from the underperforming low and medium doses. Simultaneously, it maintained allocation to placebo patients to account for temporal trends in epidemiology and treatment. This approach could increase efficiency of trials by minimizing “wasted” allocation of participants to ineffective treatment arms. In our trial, this adaptive randomization maximized the use of limited resources and represents a valid strategy to consider in subsequent trials of critically ill populations.

We believe it is important to carefully consider the interpretation of the observed study outcome, specifically the predicted probability of success of a subsequent trial. Our calculation of predictive probability was conditional on meeting the SOFA score change threshold, which did not occur. We would naturally view the predictive probability of success for a new phase 3 trial to be low; however, without meeting this threshold, the reported predictive probabilities should be interpreted skeptically because they may have overestimated the probability of success in a phase 3 trial compared with a model using assumptions closer to the observed change in the SOFA scores.

### Limitations

This trial has several additional limitations to consider. We chose change in the SOFA scores as a surrogate outcome based on strong correlations between this measure and 28-day mortality.^33^ Whether change in the SOFA scores and the timing of reassessment (48 hours in this case) represents the “right” surrogate end point for nonpivotal sepsis trials remains unclear and is an area for future consideration, although the use of change in the SOFA score as a surrogate outcome is supported by a recent meta-analysis.^34^ While large for phase 2 trials in sepsis, we enrolled 250 patients, with only 106 enrolled at the highest levocarnitine dose. As such, smaller treatment effects may exist that could have been detected in a larger study, although our study was adequately powered to detect a change in the SOFA scores of 2, which was considered the minimal clinically significant change a priori. APACHE II scores were lower in the high-dose arm and may have contributed to the reduced mortality observed, although from a binary standpoint, this hypothesis does not significantly affect study interpretation given the negative results. While our study was designed and begun before release of the Third International Consensus Definitions for Sepsis and Septic Shock (Sepsis-3),^2^ the SOFA score and lactate level were considered in the enrollment criteria, so our results retain validity in the context of the updated definition. Due to the inclusion of patients with hospital-acquired infection, determination of a consistent definition for the time of sepsis onset for calculation of the time to antibiotic use was not possible, so we were unable to ensure the balancing of this factor between treatment groups. However, as an unmeasured confounder, this variable should be addressed by the randomization procedure. The specific effect of levocarnitine on metabolism among patients in sepsis was not evaluated and remains an area of future investigation.

## Conclusions

In this dose-finding, phase 2 adaptive randomized trial, patients with septic shock and moderate organ dysfunction were treated early in the course of illness with low (6 g), medium (12 g), or high (18 g) doses of levocarnitine or an equivalent volume of saline placebo administered as a 12-hour infusion. None of the tested doses of levocarnitine meaningfully reduced cumulative organ failure at 48 hours.

## Notes

Supplement 1. **Trial Protocol**

Supplement 2. **Data Sharing Statement**
