# Supplementary material for: Effect of Levocarnitine vs Placebo as an Adjunctive Treatment for Septic Shock: The Rapid Administration of Carnitine in Sepsis (RACE) Randomized Clinical Trial
Source: JAMA Netw Open. 2018 Dec 21;1(8):e186076. doi: 10.1001/jamanetworkopen.2018.6076 (PMC6324339; doi:10.1001/jamanetworkopen.2018.6076)
Supplement: Supplement 1. — Trial Protocol [file jamanetwopen-1-e186076-s001.pdf]

MANUAL OF PROCEDURES AND STUDY PROTOCOL

STUDY TITLE: **R**apid **A**dministration of **C**arnitine in s**E**psis (**RACE**)

STUDY DRUGS: Levocarnitine (L-carnitine)

PRINCIPAL INVESTIGATOR
Alan E. Jones, MD
University of Mississippi Medical Center
Department of Emergency Medicine
2500 North State Street
Jackson, MS 39216
aejones@umc.edu

SITE CO-INVESTIGATORS:

Michael Puskarich, MD
Assistant Director of Research
Department of Emergency Medicine
University of Mississippi Medical Center
Jackson, MS

Stephen Trzeciak, MD, MPH
Director of Research
Department of Medicine
Cooper University Hospital
Camden, NJ

Jeffrey A. Kline, MD
Director of Research
Department of Emergency Medicine
Indiana University School of Medicine
Indianapolis, IN

Jason Adams, MD
Associate Professor
Department of Emergency Medicine
University of California, Davis
Sacramento, CA

Nathan I. Shapiro, MD, MPH
Director of Research
Department of Emergency Medicine
Beth Israel Deaconess Medical Center
Boston, MA

Robert Sherwin, MD
Director of Research
Department of Emergency Medicine
Wayne State University
Detroit, MI

Michael Runyon, MD
Director of Research
Department of Emergency Medicine
Carolinas Medical Center
Charlotte, NC

Ryan Arnold, MD
Department of Emergency Medicine
Christiana Care Health Services
Wilmington, DE

D. Mark Courtney, MD, MSCI
Director of Research
Department of Emergency Medicine
Northwestern University
Chicago, IL

Michael Kurz, MD
Associate Professor
Department of Emergency Medicine
University of Alabama at Birmingham
Birmingham, AL

Faheem W. Guirgis, MD
Assistant Professor
Department of Emergency Medicine
University of Florida College of Medicine
Jacksonville, FL

Michael Filbin, MD
Assistant Professor
Department of Emergency Medicine
Massachusetts General Hospital
55 Fruit Street
Boston, MA 02114-2696

Peter Hou, MD
Associate Physician
Department of Emergency Medicine
Brigham and Women's Hospital
75 Francis Street
Boston, MA 02115-6110

<u>DATA SAFETY MONITORING BOARD:</u>

Chair: Roger Lewis, MD, PhD

Members: Don Yealy, MD
Jill Baren, MD

Statistician: Christopher Coffey PhD

TABLE OF CONTENTS

Section		Page
1.0	INTRODUCTION	4
2.0	OBJECTIVES AND SPECIFIC AIMS	4
3.0	BACKGROUND AND SIGNFICANCE	6
4.0	RESEARCH DESIGN AND METHODS	12
	A. TRIAL DESIGN	12
	B. TRIAL SITES	12
	C. STUDY SETTING AND POPULATION	12
	D. INCLUSION CRITERIA	13
	E. EXCLUSION CRITERIA	13
	F. TREATMENT ARMS	14
	G. RISK BENEFIT RATIO	14
	H. STUDY PROCEDURES	15
	1) SCREENING AND ENROLLMENT	15
	2) STUDY INTERVENTIONS	16
	3) ENDPOINTS AND DATA COLLECTION	18
	4) ADVERSE EVENT REPORTING	19
5.0	TRIAL STRATEGY AND STATISTICAL ANALYSIS	23
6.0	TRIAL OVERSIGHT	26
7.0	REFERENCES	33

1.0 INTRODUCTION

Study Director and Principal Investigator: Alan Jones, MD

Study Sites: University of Mississippi Medical Center, Beth Israel Deaconess Medical Center, Cooper University Hospital, Indiana University Medical Center, Northwestern University Medical Center, Carolinas Medical Center, University of California at Davis, Wayne State University, Christiana Care Health Services, University of Alabama at Birmingham, University of Florida College of Medicine at Jacksonville, Massachusetts General Hospital, Brigham and Women's Hospital

Coordinating Center: University of Mississippi Medical Center

Sponsor: National Institute of General Medical Sciences

Sponsor Contact: Sarah Dunsmore, PhD

DSMB Chair: Roger Lewis, MD, PhD

Statistical Consultant: Scott Berry, PhD, Mike Griswold, PhD

Trial Steering and Writing Committee: Alan Jones, MD, Jeffrey Kline, MD, Nathan Shapiro, MD, MPH, Stephen Trzeciak, MD, MPH

Regulatory Information: This trial will be conducted under the authority of the study director based upon Food and Drug Administration IND # 107,186. Trial oversight will be in accordance with the Code of Federal Regulations (21CFR312), Good Clinical Practice Guidelines and International Conference on Harmonisation Guidelines. This study will be registered on clinicaltrials.gov prior to enrollment and in public communication will be referred to as RACE (**R**apid **A**dministration of **C**arnitine in s**E**psis).

2.0 OBJECTIVES AND SPECIFIC AIMS

More humans die in the intensive care unit from sepsis than from any other cause. Death from sepsis is the culmination of widespread hypoperfusion, cellular hypoxia, and multiple organ failure. A growing body of evidence shows that early therapeutic intervention improves outcome for patients with sepsis. Novel targeted strategies that bolster a strong and durable systemic hemodynamic response have been proven to reduce or even reverse organ dysfunction in patients with sepsis. *L-carnitine* provides the key elements of a ***novel therapy*** to ameliorate the adverse hemodynamic

effects of sepsis. Severe physiological stress forces energy metabolism to shift from primary fatty acid oxidation toward glycolysis and lactate oxidation. Prior work has shown that exogenous L-carnitine administration enhances glucose and lactate oxidation, attenuates fatty acid toxicity and improves endothelial-smooth muscle coupling and cardiac mechanical efficiency. The overall goal of this proposal is to investigate L-carnitine as a novel adjunctive treatment of septic shock; specifically to test the clinical efficacy of L-carnitine on reducing organ failure, a critical patient oriented outcome, and provide evidence as to whether stimulation of carbohydrate oxidation creates host resilience to severe sepsis.

Clinical Efficacy Hypothesis 1: Early adjunctive L-carnitine administration in vasopressor dependent septic shock will significantly reduce cumulative organ failure at 48 hours with an associated decrease in 28-day mortality suggesting the need for further phase III study.

Specific Aim 1: **Test if intravenous L-carnitine reduces cumulative organ failure in septic shock.**

SA1a. Using an adaptive trial design, randomize up to 250 eligible patients with septic shock to receive low (6g), medium (12g) or high (18g) dose intravenous L-carnitine or placebo for 12 hours.

SA1b. Measure the first efficacy endpoint of reduction in cumulative organ failure, defined as a decrease in the sequential organ failure assessment (SOFA) score at 48 hours after treatment, and use the SOFA data to preferentially allocate subsequent patients to the L-carnitine dose that is most effective.

SA1c. Measure the second efficacy endpoint of mortality at 28 days, and determine in an ongoing manner as the trial progresses, the probability that the dose of L-carnitine associated with the largest decrease in SOFA score would demonstrate efficacy in a subsequent phase III, randomized, placebo-controlled trial.

Rationale: L-carnitine enhances lactate oxidation, which enhances cardiac inotropy and vascular smooth muscle tone, thus enhancing vital organ perfusion.

Expected results: Significantly more patients treated with the optimal dose of L-carnitine will demonstrate a decrease SOFA score as compared to placebo and this dose will have a substantial probability of success in a follow up phase III trial with a mortality endpoint.

Clinical Mechanism Hypothesis 2: L-carnitine will produce a significant improvement in microcirculatory blood flow in patients with vasopressor dependent septic shock.

Specific Aim 2: **Test if L-carnitine improves blood flow in the sublingual microvasculature during septic shock.**

SA2a. Perform sidestream dark-field (SDF) video-microscopy of the sublingual microcirculation, prior to and after 12 hours of L-carnitine or placebo. The outcome is the change in microcirculatory flow index (ΔMFI).

Rationale: L-carnitine should attenuate or reverse vasoplegia, one of the main causes of microcirculatory insufficiency in septic shock. The ΔMFI will provide insight into the clinical mechanism of L-carnitine benefit. *Expected results*: L-carnitine patients will have a significant improvement in MFI compared to placebo.

3.0 BACKGROUND AND SIGNIFICANCE

Sepsis is a significant public health problem. Sepsis is common. It is estimated that severe sepsis occurs at an incidence rate of 3 cases per 1,000 persons/year, resulting in approximately 750,000 affected persons annually in the U.S. Of those affected, 500,000 (67%) require intensive care unit (ICU) services.[1] Sepsis is growing. Recent estimates indicate that the rate of severe sepsis hospitalizations doubled during the last decade, and its prevalence exceeds that of other high profile diseases in the U.S. During 2002, for example, the number of cases per 100,000 population of severe sepsis far exceeded the number of cases of acquired immunodeficiency syndrome (AIDS), venous thromboembolism (VTE) and both colon and lung cancer.[1-4] Furthermore, the incidence of severe sepsis is projected to increase by 1.5% per annum, resulting in more than 1 million cases per year by 2020.[1] Sepsis kills. Sepsis ranks as the tenth leading cause of death in the U.S., resulting in 215,000 deaths annually.[1;5] In the U.S., with a hospital mortality rate of 30%, mortality rates due to sepsis are strikingly higher than other high profile diseases such as stroke, myocardial infarction, and trauma.[1;6-8] Moreover, the highest mortality rate from sepsis occurs among our nation's elderly, those who are 65 years or older, and age-adjusted population based mortality is increasing.[1;9] As the U.S. population ages, sepsis will remain a massive problem. Sepsis costs. The total annual cost to the U.S. directly attributable to this disease is $16.7 billion, representing a substantial demand on hospital resources including prolonged occupancy, specialty equipment, and staff.[1]

The investigation of L-carnitine addresses an important knowledge gap in sepsis therapeutics. Two reasons for the high mortality rates of septic shock are the damage sepsis imparts on the cardiovascular and microvascular systems and our current limitations in ameliorating that damage. Presently, no therapeutic agent reverses the cardiovascular and microvascular derangements caused by septic shock. As a result, clinicians must react to manifestations of septic shock by using supporting treatments. In reaction to hypotension, clinicians infuse fluids, vasopressors and inotropic agents to support the cardiovascular system. While these agents can augment blood pressure, vasopressors can increase oxygen demand and paradoxically increase net tissue hypoxia, while inotropic agents can induce adverse side effects such as dysrhythmias and intolerable hypotension. Moreover, the efficacy of all catecholamine derivatives are limited by the adverse effect of tachyphylaxis. A substantial body of literature supports the assertion that L-carnitine can reliably and safely support the cardiovascular system in sepsis without these adverse effects. *If we identify a new treatment for sepsis that is both commonly available and has an important impact on patient-oriented outcomes, then scientific knowledge would be improved and clinicians would gain a powerful agent in their armamentarium for the management of severe sepsis in the acute phase.*

Carnitine is a naturally occurring nutrient required in mammalian energy metabolism. It has been shown to facilitate long-chain fatty acid entry into cellular mitochondria, therefore delivering a substrate for oxidation and subsequent energy production. *L*-carnitine refers to the *levo* isomer of the compound, which is biological active. It is obtained primarily from dietary intake of protein, though it can be synthesized in the liver and kidneys from the amino acids lysine and methionine. Although it is present throughout the body in mitochondria, L-carnitine is concentrated in muscle tissue, including the heart, skeletal and smooth muscle, as well as the endothelial cell layer.

Figure 1. Role of carnitine in energy metabolism.

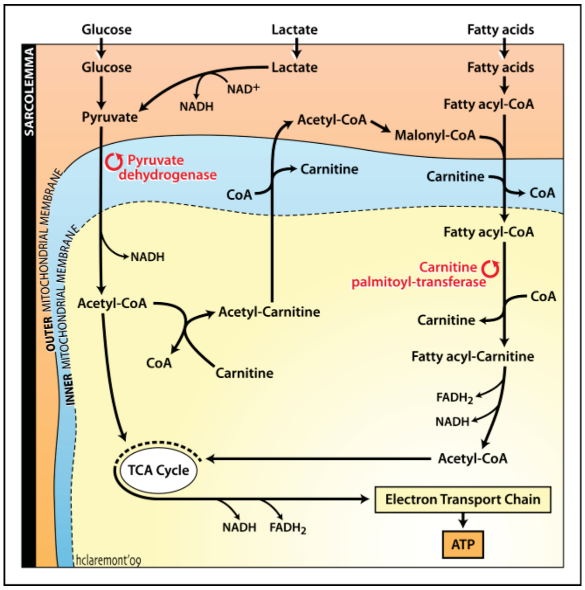

Healthy heart muscle uses a combination of free fatty acids (FFA), glucose, and lactate for energy, with FFAs providing 50-70% of the energy for the myocardium.[10;11] As depicted in **Figure 1**, FFAs must be esterified to L-carnitine to be capable of crossing the mitochondrial membranes via the tandem action of L-carnitine palmatoyl transferases I and II (CPT-I, II).[12] Key to the study hypothesis, L-carnitine also modulates activity of the

matrix enzyme pyruvate dehydrogenase (PDH), which comprises three enzymatic components: decarboxylation, dihydrolipamide acetyltransferase, and dihydrolipoamide dehydrogenase (E1, E2, and E3, respectively). The PDH complex performs oxidative decarboxylation of pyruvate, a key rate-limiting step, required for lactate disposal. The E1 component of the PDH complex is inhibited by phosphorylation of its active site by a PDH kinase, which is stimulated by an increased acetyl-CoA/CoA ratio. The E1 is activated by a Ca^{++}- and insulin-stimulated phosphatase that dephosphorylates serine and threonine residues in its active site. L-carnitine accelerates pyruvate oxidation (and the forward flow of lactate disposal) by esterifying and exporting acetyl-CoA out of the mitochondrial matrix, thereby decreasing the acetyl-CoA/CoA ratio.[39;13] Through this mechanism L-carnitine infusion has been demonstrated not only to improve FFA but also carbohydrate metabolism.[14,15] These roles as an essential component at *rate-limiting steps* of combustion of multiple fuel substrates make L-carnitine an attractive therapeutic target for diseases that feature derangements of metabolism.

During stress conditions, such as sepsis, the myocardium drastically alters its primary source of energy from FFAs to lactate, as illustrated in **Figure 2.**[10;11] Septic shock also induces profound inefficiencies in myocardial work not explained by cellular or mitochondrial hypoxia.[16;17] Although the mechanisms of this inefficiency are not completely understood, research has shown that the cardiac isozyme of CPT-I (the key mediator of FFA transport) is significantly down-regulated in sepsis, contributing to decreased FFA metabolism.[18-21] Simultaneously, excessive acetyl-CoA production stimulated by sepsis alters the acetyl-CoA/CoA ratio and inhibits PDH.[13;22] This imbalance leads to decoupling of the stoichiometry between glycolysis and pyruvate oxidation. Consequently, the cytosol accumulates lactate, FFA metabolites, and intracellular protons, which together exert a tremendous intracellular cost in terms of chemical energy required to remove them.[15;23] Inhibition of both of these enzymes in sepsis decreases the primary supplies of energy to the failing heart, and likely plays a role in sepsis-induced cardiac dysfunction.

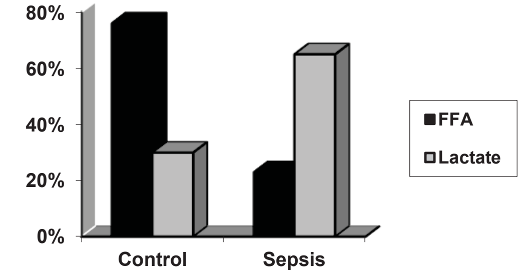

Figure 2. Cardiac fuel in sepsis

During septic shock the cardiovascular system experiences a net loss of tissue L-carnitine, both in the myocardium[24] and endothelium.[25] L-carnitine is redistributed from the tissue to the peripheral blood, resulting in transiently elevated plasma L-carnitine levels.[26] The L-carnitine resorption mechanism in

the kidney, which demonstrates saturation kinetics[27] and is highly efficient (90-99%) in healthy individuals, is overwhelmed by the increased plasma levels of L-carnitine in sepsis, resulting in increased urinary excretion, which in turn ultimately leads to whole body L-carnitine loss.[28;29] Importantly, L-carnitine deficiency has been demonstrated to result in impaired cardiovascular function in both septic and non-septic conditions.[39;30;31] **Exogenous infusion of L-carnitine effectively increases L-carnitine levels in depleted tissues in animal models of sepsis[32;33]** and enhances cardiac contractility in depressed hearts primarily by stimulating pyruvate oxidation, recoupling glycolysis and oxidation of pyruvate, leading to more efficient heart muscle contraction in shock.[34-36] Improvement in carbohydrate and lactate metabolism, which are more efficient than FFAs in energy production, is a primary reason for L-carnitine's ability to improve cardiac efficiency in states of stress.[23] Therefore, as opposed to vasoactive agents, which increase the amount of cardiac work, ***L-carnitine functions as a metabolic therapy, improving the efficiency of cardiac work.***

Like the rest of the cardiovascular system, the vascular endothelium utilizes L-carnitine-dependent FFA oxidation.[25;37] Elevated levels of serum FFA, a common finding in sepsis, disrupts the chemical signaling between the endothelial cell layer and smooth muscle cells, contributing to vasoplegia.[38;39] The endothelium appears to be particularly vulnerable to the intermittent ischemia associated with altered microcirculatory flow, and leads to preferential loss of low molecular weight compounds, including L-carnitine.[25;40] Animals deficient in L-carnitine demonstrate exaggerated vasoplegic response to a LPS challenge.[30] Infusion of L-carnitine can lead to restoration of flow regulation after periods of intermittent ischemia, an effect that may be related to changes in endothelial permeability.[37;41] Additionally, L-carnitine has been shown to improve endothelial function through and antioxidant effect of decreasing superoxide anion production and increasing nitric oxide participation.[42] As microcirculatory dysfunction is a key mechanism in the evolution of septic shock, and L-carnitine improves endothelial function, directly imaging the microcirculation via intra-vital video-microscope technology will provide us an opportunity to explore a key mechanism of a potential therapy for sepsis-induced organ failure.

Preliminary human data suggests that L-carnitine infusion in septic shock improves patient oriented outcomes. One previous study examined the effect of L-carnitine infusion on patients with septic shock.[43] Gasparetto et al. randomized 115 patients with circulatory shock to receive either 12 grams of L-carnitine over 12 hours or placebo. Of these 115 patients, 72 had sepsis as their etiology of shock. Among the 72 septic shock patients, <u>those that received L-carnitine had significantly higher</u>

systolic and mean arterial pressures, lower right atrial pressure, and higher arterial partial pressure of oxygen and hemoglobin oxygen saturation at the end of the L-carnitine infusion as compared to placebo treated patients. These results suggest the potential for L-carnitine to improve dysfunction of multiple organ systems in septic shock and provide an important basis for the use of change in cumulative organ failure as a primary outcome measure.

Microcirculatory dysfunction is a pivotal element in the pathogenesis of severe sepsis and septic shock. Microcirculatory dysfunction is a pivotal element in the pathogenesis of severe sepsis and septic shock.[44-47] Using intra-vital video-microscopy, experimental models of sepsis have demonstrated decreased microcirculatory flow velocity, "stopped-flow" microvessels, increased heterogeneity of regional perfusion, and decreased density of perfused capillaries.[48-50;50;51] These derangements can cause marked alterations of oxygen transport including impaired tissue oxygen delivery and consumption leading to anaerobic cellular metabolism.[52] With the advent of new imaging modalities such as sidestream dark field (SDF) video-microscopy, it is now possible to visualize the microcirculatory network in human subjects. *Microcirculatory "failure" is one of the critical pathogenic events in sepsis that is associated with acute multi-organ dysfunction and mortality.*[44-46] Although septic shock research is classically focused on macrocirculatory hemodynamics that reflect the distribution of blood flow globally throughout the body, a functioning microcirculation is another critical component of the cardiovascular system that is essential for *effective* oxygen delivery to tissues.

The SDF microcirculatory imaging technique utilizes optical filtration of polarized light that is absorbed by hemoglobin so that red blood cells appear dark. This permits direct visualization of blood flow in the sublingual microcirculatory network in human subjects in a non-invasive fashion using a hand-held video-microscope (**Figure 3**). The technique has been validated in both experimental and human studies.[53-55] Several investigators have demonstrated that impaired sublingual perfusion can track impairment of splanchnic perfusion and detect early systemic perfusion failure in shock states.[56-59] Monitoring sublingual blood flow can yield important information for use in clinical studies of circulatory shock because (1) the sublingual mucosa shares the same embryologic (and therefore anatomic) origin as the splanchnic mucosa, (2)

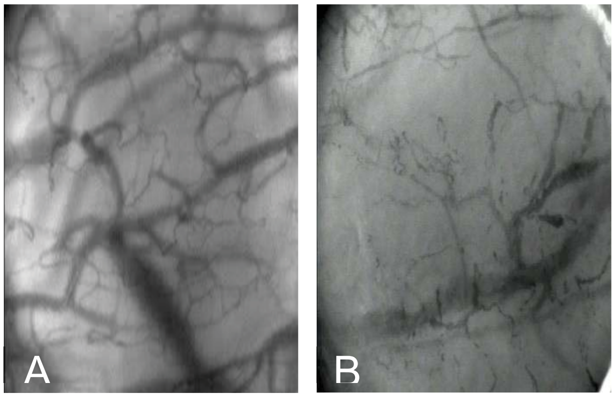

Figure 3. *Image A* - Normal microcirculation. *Image B* - Microcirculation in a patient with septic shock. Note the lack of perfused vessels in the sepsis (image B) compared to the normal (image A).

derangements in sublingual perfusion reflect derangements in splanchnic blood flow [56;57;59-62] and (3) the sublingual space is easily accessible. Because splanchnic hypoperfusion can be one of the earliest indicators of systemic hypoperfusion in circulatory shock,[63] impaired sublingual blood flow can herald the onset of systemic hypoperfusion.[58;59] In the proposed randomized controlled trial, we will use SDF videomicroscopy to determine whether a novel metabolic therapy for sepsis can augment microcirculatory flow, potential providing a mechanistic investigation of any perceived clinical effect.

Organ failure causes mortality and is a critical patient oriented research outcome measure. Acute multi-organ dysfunction is a critical event in the pathogenesis of sepsis, and is closely linked with mortality.[1;64] *Early* evidence of organ failure is an especially strong mortality predictor.[64;65] *Early* improvement in organ function (i.e., 0-48 hour improvement in the Sequential Organ Failure Assessment (SOFA) score[66] is closely related to sepsis survival,[67] whereas later improvements have little predictive value.[65] Thought leaders have *advocated the use of SOFA score as an important outcome measure for sepsis clinical trials*[68;69] and have hypothesized that microcirculatory flow impairment is a pivotal element in the pathogenesis of sepsis-induced organ failure.**[70;71]** We recently reported our experience of the utility of the SOFA score for assessing outcome of patients with severe sepsis.[72] **Figure 4** shows the relationship between baseline SOFA, 48hr SOFA change, and 28-day mortality in 246 patients with inclusion criteria identical to the proposed trial and obtained from a multicenter observational study. All patients were treated according to the Surviving Sepsis Campaign Guidelines. The x and y axes of the plot show baseline and 48hr SOFA change scores, with black circles for patients who lived and red triangles for patients who did not live. To examine the relationship we performed a logistic regression with 48 hour SOFA change as the explanatory variable and mortality as the response. The analysis indicated 48hr SOFA change is highly statistically significant (p<0.0001) with an odds ratio of 1.35 (95% CI 1.20-1.53) indicating a 35% increased odds of mortality for each point increase in 48hr SOFA change.

Figure 4. Relationship between both baseline and 48 hour delta SOFA score and 28-day mortality.

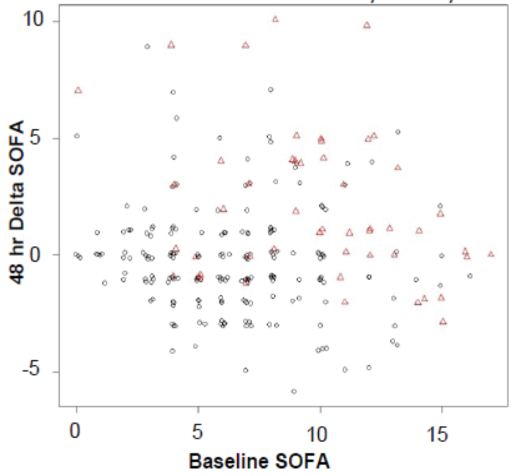

Although the use of surrogate outcomes is standard in phase II studies, we recognize their lesser clinical significance compared with a mortality endpoint. As has been noted in many previous sepsis trials, positive findings using surrogate outcomes in phase II studies have failed to translate into

meaningful outcomes (i.e. mortality rates). The SOFA score represents a strong independent predictor of 28-day mortality. As such it will be used to preferentially allocate patients to the L-carnitine dose that is most effective during the trial. The definition of success at the conclusion of the currently proposed phase II trial requires ***BOTH demonstration of improvement in SOFA score in the optimal dose intervention subjects AND the predicted probability of success of >30% in a subsequent phase III trial of the optimal dose versus placebo investigating 28-day mortality***. Using this methodology, the optimal dose will be identified and only carried to phase III if the results are promising.

4.0 RESEARCH DESIGN AND METHODS

A. Trial Design:

This trial will be a randomized, double-blind, placebo-controlled, dose/efficacy-finding trial that utilizes a Bayesian adaptive approach, incorporating a flexible dose-response model, frequent interim analyses to allow the efficient identification of a clinically-important efficacy or trial futility, and a response-adaptive randomization to increase the scientific efficiency of the trial.

B. Trial Sites:

University of Mississippi Medical Center, Beth Israel Deaconess Medical Center, Cooper University Hospital, Indiana University Medical Center, Northwestern University Medical Center, Carolinas Medical Center, University of California at Davis, Wayne State University, Christiana Care Health Services, University of Alabama at Birmingham, University of Florida College of Medicine at Jacksonville, Massachusetts General Hospital, Brigham and Women’s Hospital

C. Study Setting and Population:

Patients will be identified in the ED or ICU. Potential participants will be identified as adult patients who are treated with a quantitative resuscitation protocol for septic shock initiated in the ED or ICU within 24 hours of enrollment. The following inclusion and exclusion criteria will apply:

D. Inclusion criteria:

1) Suspected or confirmed infection (examples include but are not limited to: white cells in a normally sterile body fluid; perforated viscus; radiographic evidence of pneumonia in clincal

symptoms; a syndrome associated with a high risk of infection e.g. cellulitis, cutaneous abscess, ascending cholangitis, toxic shock syndrome, fever of unknown orgin with high suspicion of infectious etiology)

2) Any two of four criteria of systemic inflammatory response as defined by the 2001 ACCP/SCCM Consensus Conference Committee;
3) Recognition of septic shock and initiation of quantitative resuscitation within 24 hours of enrollment;
4) Requirement of high dose vasopressors to treat shock for ≥4 hours and at the time of enrollment in the study: Norepinephrine > 0.05mcg/kg/min; dopamine >10mcg/kg/min; Phenylephrine >0.4 mcg/kg/min; epinephrine > 0.05 mcg/kg/min; vasopressin = any dose
5) Cumulative sequential organ failure assessment (SOFA) score of ≥ 6;
6) Blood lactate level of >2.0 mMol/L.

E. Exclusion criteria:

1) Age <18 years;
2) Pregnancy or breastfeeding;
3) Any primary diagnosis other than sepsis;
4) Established Do Not Resuscitate status or advanced directives restricting aggressive care or treating physician deems aggressive care unsuitable;
5) Any history of seizures or a known seizure disorder;
6) Any known inborn error of metabolism;
7) Anticipated requirement for surgery that would interfere with the 12 hour infusion time;
8) Active participation in another interventional study;
9) Cardiopulmonary resuscitation (chest compression or defibrillation) prior to enrollment;
10) Known systemic allergy to L-carnitine.
11) Severe immunocompromised state (e.g. subject has neutropenia [receiving cytotoxic chemotherapy with absolute neutrophil count <500/uL or expected to decline to < 500 uL within the next three days).
12) Active coumadin treatment

F. Treatment Arms:

The trial will include one control and three active treatment arms. The control (placebo) arm is a normal saline solution. The treatment arms are L-carnitine in three different doses: low (6 grams), medium (12 grams) and high (18 grams), each administered intravenously over a 12-hour period. Neither the investigators nor the research participants will know to which arm the participant has been assigned. The pharmacist who mixes the treatment solution will not be blinded; however, this individual will have no study-related contact with the investigators or the study participants.

Selection bias should not be an issue given that the investigator is blinded from group assignments and from the randomization procedure itself. The research pharmacists and staff are the only study personnel who have access to the group assignment given to each subject. To ensure blinding, the pharmacist will prepare either L-carnitine or placebo in identical polypropylene infusion bags and label the bags with identical labels that will include the patient's study ID number, patient name, medical record number, and infusion rate. For each dose of L-carnitine, 33% of the total dose will be administered as a bolus over 2-3 minutes followed by a fixed rate continuous infusion over the next 12 hours. For example in the medium dose (12 gram) arm the patient would receive a 4 gram bolus injection over 2-3 minutes followed by 8 gram infusion (8 grams in 1000 ml of 0.9% normal saline) over the following 12 hours (83 ml/hr). The placebo groups will have the identical number and appearance of polypropylene infusion bags filled with normal saline and run at the same rates as the active treatment arms. The bags will not otherwise be identified. The study solution will be transported to the patient's care area where it will be administered by the patient's clinical care nurse and supervised by the site PI. The study solution will be administered via an existing central venous catheter, placed as a part of each institution's early quantitative resuscitation protocol, and will be administered using FDA-approved medical equipment (IV tubing, IV pumps, etc.) that is routinely available at study sites. Sigma-Tau Pharmaceuticals, Inc. will provide the study drug, which will be maintained by the pharmacy staff. A standard operating procedure will be used to track lot numbers of L-carnitine administered. The pharmacy will keep a drug accountability log.

G. Risk/Benefit Ratio:

Patients eligible for enrollment have at least a 40%-50% probability of experiencing death in the hospital and as much as a 25% chance of experiencing a serious side effect from vasopressors (supraventricular or ventricular arrhythmias, acute coronary event, limb or penile necrosis, stroke, CNS bleeding), representing substantial risk of morbidity or mortality from the disease process. L-

carnitine has a very favorable safety profile, with no known significant or serious side effects. All adverse effects that have previously been reported are mild such as abdominal cramping and body odor or moderate such as increase seizure risk. If L-carnitine infusion is able to reduce the amount of organ dysfunction, this might directly result in improved chance for survival and decreased hospital length of stay and its potential complications.

H. Study Procedures:

1) **Screening and Enrollment**: All of the trial sites have an established protocolized quantitative resuscitation strategy for septic shock. Site PIs will assure that research personnel are always available to cover this study 24 hours a day, 7 days a week. The inclusion criteria require enrollment within 24 hours of identification and initiation of quantitative resuscitation for septic shock and the use of high dose vasopressors for 4 or more hours. These criteria allow a reasonable window for enrollment but still satisfy the aim of the study of early administration of L-carnitine therapy. A timeline for study enrollment is shown in **Figure 5**. Eligible patients (or their surrogate in the case of mental incapacitation) will be approached for consent.

Figure 5

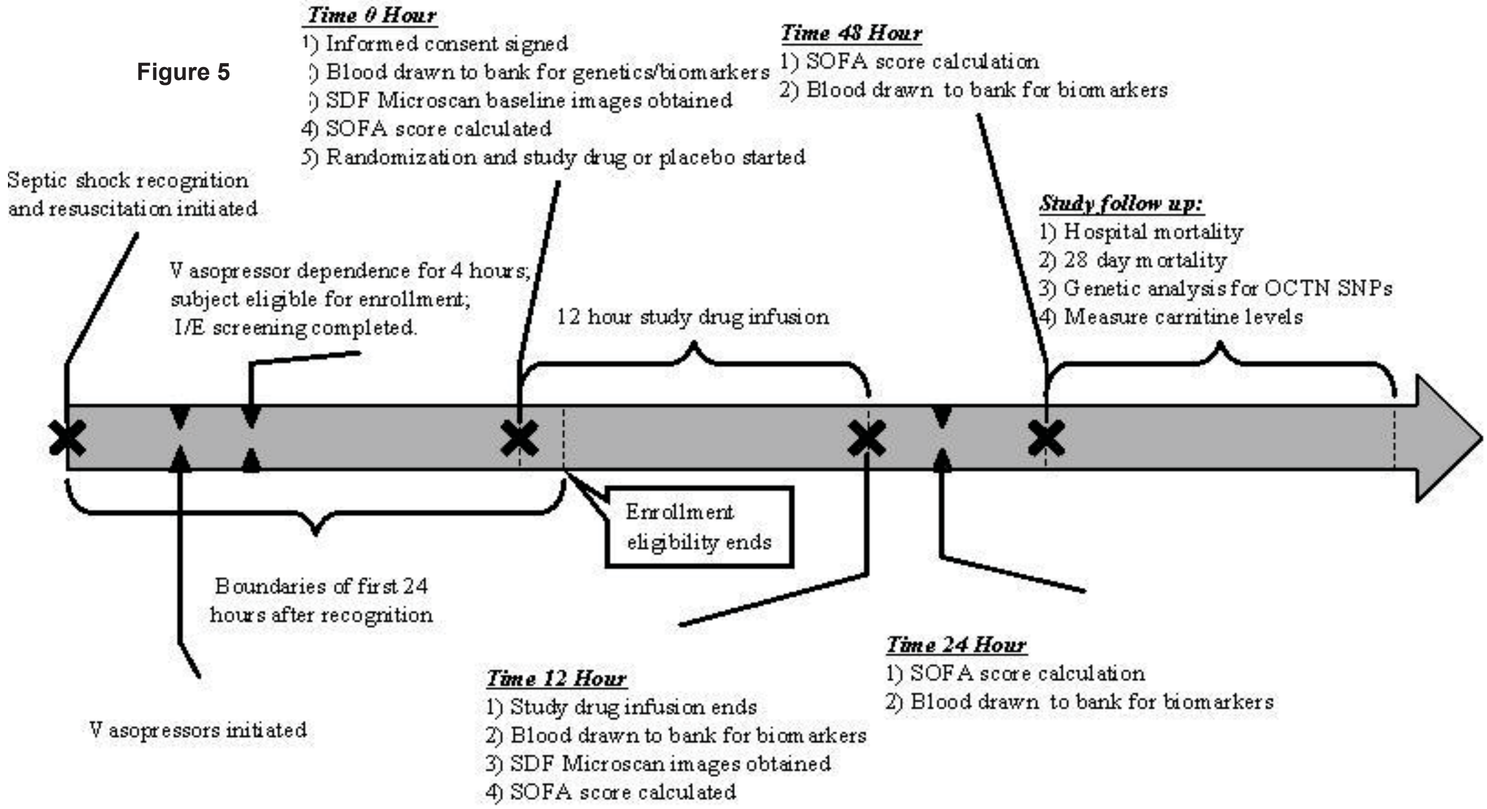

2) **Study Interventions**: After enrollment the following study procedures will occur (Figure 5)

   a. Pre-treatment (Pre- T0) and Time 0 (T0)
      i. Determination that study entry inclusion and exclusion criteria have been met
      ii. Physical examination and assessment of disease severity (e.g., APACHE II)
      iii. Randomization;
      iv. Blood draw for biomarkers and genetics;
      v. SOFA score calculation (mandatory all data included-mandatory SOFA score for T0 to be calculated using data at T0 ± 4 hours;
      vi. Microscan of the sublingual microcirculation;
      vii. Study drug initiation (T0)
      i through iv and vi have to be performed PRIOR to vii

   b. Time 12 (T12 ± 4 hours)
      i. Physical examination
      ii. Vital signs
      iii. New medications and therapeutic interventions
      iv. Occurrence of adverse events
      v. Blood draw for biomarkers;
      vi. SOFA score calculation;
      vii. Microscan of the sublingual microcirculation;
      viii. Study drug infusion ends approximately 12 hours after the start of infusion

   c. Time 24 (T24 ± 4 hours)
      i. Physical examination
      ii. Vital signs
      iii. New medications and therapeutic interventions;
      iv. Occurrence of adverse events;
      v. Blood draw for biomarkers;
      vi. SOFA score calculation;

d. Time 48 (T48 ± 4 hours)
    i. Physical examination
    ii. Vital signs
    iii. New medications and therapeutic interventions
    iv. Occurrence of adverse events (48h-7days);
    v. Blood draw for biomarkers (48 and 72 hours);
    vi. SOFA score calculation (mandatory all data included- mandatory SOFA score for T48 to be calculated using data at T48 ± 4 hours);

e. Hospital discharge
    i. Vital Status

f. 28-day
    i. Phone call for vital status

g. 3 month, 6 month and 1 year mortality
    i. Social Security Death Index search for vital status

Routine Labs: All patients enrolled in the study are critically ill and as such laboratory studies are routine in such patients. Patients should have the following laboratory assessments collected at baseline, at 12 hours after therapy (T24 hours), and 36 hours after therapy (T48 hours): CBC with differential, blood chemistry, liver function tests, PT, PTT, urinalysis. Additionally a baseline EKG will be performed and recorded. All women will have a urine or serum pregnancy test performed prior to drug administration.

Biomarkers: All enrolled subjects will have serial draws for future measurements of blood content of key components of the inflammatory cascade as well as carnitine levels. In general the measurements will be done as follows: up to 4 hours prior to L-carnitine initiation (T0), at the completion of the infusion (T12 ± 4 hours), at T24 hours ± 4 hours, and, T48 hours ± 4 hours, and T72 hours ± 4 hours. All blood draws will be performed via access of the central venous catheter, where available. Approximately, 45 cc of blood will

be required at 5 time points. Using an SOP, plasma will be collected, centrifuged and stored. All stored will be batched and shipped to the coordinating center for storage.

Genomic variations: As genomic variations in the carnitine transporter may affect the results of our study, we will collect a portion of the 45 cc of blood drawn at the T0 time point outlined above in a Paxgene tube to allow DNA and RNA recovery. This will allow for subsequent analysis of sequence variations, expression profiles, and allow for correlation to observed phenotypes, including responders versus non-responders.

Microscan:. We will visualize the sublingual microcirculation with an FDA exempt Incident Dark Field (IDF) video microscope (Braedius Medical, BV, Huizen, The Netherlands) with a field of view of approximately 1.5 mm x 1 mm (width x height). The IDF probe will be placed in the sublingual space and we will obtain video sequences of 10 seconds each from five different sublingual sites. Images are recorded by the Cytocam Tools program (Braedius Medical, BV, Huizen, The Netherlands) on a dedicated computer workstation. The images are either transferred to an external drive or uploaded for data analysis. No PHI is stored on the dedicated computer workstation. No PHI is transferred.
Images will be obtained at T0 (up to 4 hours prior to carnitine administration) and a second time, at T12 (± 4 hours) after carnitine administration is initiated. The video microscope is non-invasive and is not harmful in any way other than some potential mild discomfort in the mouth.

3) **Endpoints and Data Collection**: The following data for determination of endpoints will be collected. Additional data to be collected can be found on the complete enrollment collection templates and case report forms.

Primary Clinical Endpoints

- Reduction in organ failure, defined as a decrease in sequential organ failure assessment (SOFA) score from T0 to T48.
- 28 day mortality

Secondary Clinical Endpoints

- Time to vasopressor withdrawal;
- ICU care free days (number of days not requiring vasopressor or mechanical ventilation);
- ICU length of stay (LOS);
- Hospital LOS
- Organ support requirements (days of ventilatory support, dialysis, vasopressors)
- In-hospital mortality
- 3 month, 6 month and 1 year mortality: established by medical record and Social Security Death Index (SSDI) search
- Survival by severity of illness measure at enrollment (e.g., APACHE II score)

Mechanistic Endpoints

- Change in microcirculatory flow index from T0 to T12.

4) **Adverse Event Reporting**: Safety assessments will consist of monitoring and reporting adverse events (AEs) and serious adverse events (SAEs) that occur, their relatedness to L-carnitine, all events of death, and any study specific issue of concern.

Adverse Events

An AE is any unfavorable and unintended sign, symptom, or disease temporally associated with the use of L-carnitine in this study. The study-specific definition of "temporally associated" includes up to seven days after discontinuation of L-carnitine infusion.

This includes the following:

- AEs not previously observed in the subject that emerge during the protocol-specified AE reporting period.
- Preexisting medical conditions (other than the condition being studied) judged by the investigator to have worsened in severity or frequency or changed in character during the protocol-specified AE reporting period.

- Abnormal laboratory values that fall into an abnormal range based upon the hospital's laboratory standards, the abnormality was not preexisting prior to enrollment, and the abnormality leads to a new treatment or ongoing treatment within the AE time frame.

Serious Adverse Events

An AE should be classified as an SAE if:

- It results in death (i.e., the AE actually causes or leads to death).
- It is life threatening (i.e., the AE, in the view of the investigator, places the subject at immediate risk of death. It does not include an AE that, had it occurred in a more severe form, might have caused death).
- It requires or prolongs inpatient hospitalization.
- It results in persistent or significant disability/incapacity (i.e., the AE results in substantial disruption of the subject's ability to conduct normal life functions).
- It results in a congenital anomaly/birth defect in a neonate/infant born to a mother exposed to the investigational product.
- It is considered a significant medical event by the investigator based on medical judgment (e.g., may jeopardize the subject or may require medical/surgical intervention to prevent one of the outcomes listed above).

Methods and Timing for Assessing and Recording Safety Variables

The investigator is responsible for ensuring that all AEs and SAEs that are observed or reported during the study are collected and reported to the FDA and appropriate IRB(s) in accordance with CFR 312.32 (IND Safety Reports).

Adverse Event Reporting Period

The reporting period for all study-defined adverse events will begin at the time of L-carnitine infusion and for 7 days after infusion. Patients will be followed for SAEs until hospital discharge and/or the 28-day follow up phone call. Every reasonable attempt will be made to follow laboratory abnormalities until return to normalization or baseline.

Assessment of Adverse Events

All AEs whether volunteered by the subject, discovered by study personnel during questioning, or detected through physical examination, laboratory test, or other means will be reported up until 7 days after L-carnitine infusion completion. Abnormal laboratory values will be considered an AE if the value falls into an abnormal range based upon the hospital's laboratory standards, the abnormality was not preexisting prior to enrollment, and the abnormality leads to a new treatment or ongoing treatment within the AE time frame.

Each reported SAE will be described by its duration (i.e., start and end dates), regulatory seriousness criteria if applicable, suspected relationship to L-carnitine and actions taken.

To ensure consistency of SAE causality assessments, investigators will apply the following general guideline:

Yes – There is a plausible temporal relationship between the onset of the AE and administration of the L-carnitine and the AE cannot be readily explained by the subject's clinical state, intercurrent illness, or concomitant therapies; and/or the AE follows a known pattern of response to L-carnitine or the AE abates or resolves upon discontinuation of L-carnitine.

No – Evidence exists that the AE has an etiology other than the L-carnitine (e.g., preexisting medical condition, underlying disease, intercurrent illness, or concomitant medication); and/or the AE has no plausible temporal relationship to L-carnitine.

Sepsis Related Expected Events

The following signs, symptoms, observations and events are frequently observed in association with sepsis. Such events will still be reported but the expected event list can serve as a guide when determining the relatedness to the study drug: dyspnea, chest pain, fever, hypoxemia, rapid pulse, rapid respiratory rate, dizziness, syncope, altered mental status, seizure, confusion, anxiety, generalized weakness, anorexia, nausea, abdominal pain, back pain, constipation, vomiting, pneumonia, acute renal failure, skin infection, cancer, surgery not related to treatment of sepsis, electrocardiography abnormalities (atrial arrhythmias, right bundle branch block, and ST and T wave changes), elevated troponin level, elevated BNP or NT ProBNP level, high white blood cell count, pulmonary infiltrate, pleural effusion, cardiomegaly, peri- or endocarditis, electrolyte imbalances, hypo- or hyperglycemia, need for oxygen therapy, need for vasopressor, need for blood product transfusion, need for inotropic therapy, need for mechanical ventilation, need for physical or occupational therapy.

Specific Instructions for Recording Adverse Events

- **Investigators will use correct medical terminology/concepts when reporting AEs or SAEs. Avoid colloquialisms and abbreviations.**Diagnosis vs. Signs and Symptoms: If known at the time of reporting, a diagnosis will be reported rather than individual signs and symptoms. However, if a constellation of signs and/or symptoms cannot be medically characterized as a single diagnosis or syndrome at the time of reporting, it will be reported based upon the information that is currently available. If a diagnosis is subsequently established, it will be reported as follow-up information.
- Deaths: All deaths that occur during the SAE reporting period, regardless of attribution, will be reported to the appropriate parties. When recording a death, the event or condition that caused or contributed to the fatal outcome will be reported as the single medical concept. If the cause of death is unknown and cannot be ascertained at the time of reporting, report "Unexplained Death".
- Preexisting Medical Conditions: A preexisting medical condition is one that is present at the start of the study. Such conditions will be recorded as medical and surgical history, where the case report form allows. A preexisting medical condition will be re-assessed throughout the trial and reported as an AE or SAE only if the frequency, severity, or character of the condition worsens during the study. When reporting such events, it is important to convey the concept that the preexisting condition has changed by including applicable descriptors (e.g., "more frequent headaches").
- Hospitalizations for Medical or Surgical Procedures: Any AE that results in prolonged hospitalization will be documented and reported as an SAE. If a subject is hospitalized to undergo a medical or surgical procedure as a result of an AE, the event responsible for the procedure, not the procedure itself, will be reported as the SAE. Hospitalizations for the following reasons do not require reporting: Prolonged hospitalization for diagnostic or elective surgical procedures for preexisting conditions, or hospitalization or prolonged hospitalization for scheduled therapy of the target disease of the study.

- Pregnancy: Pregnant patients will be excluded from study. In women of child-bearing age who have a uterus, pregnancy can be excluded by either urine pregnancy testing or appropriate history of abstinence, contraception with reliable menstrual history.
- Post-Study Adverse Events: The investigator will expeditiously report any SAE occurring after a subject has completed or discontinued study participation if attributed to prior L-carnitine. If the investigator becomes aware of the development of cancer or a congenital anomaly in a subsequently conceived offspring of a female subject who participated in the study, this will be reported as an SAE.
- SAE Reporting: Investigators will report all SAEs to the local IRB, data coordinating center, and the FDA (as applicable).

5.0 TRIAL STRATEGY AND STATISTICAL ANALYSIS

We propose an adaptive, phase II trial to efficiently identify the dose of L-carnitine that provides the greatest improvement in SOFA score and, simultaneously, to assess the probability of success if that dose of L-carnitine was evaluated in a confirmatory, phase III trial with a 28-day mortality endpoint. To ensure we identify the best dose, a broad range will be considered initially (6 g, 12 g, and 18 g). Because L-carnitine is likely to have its most direct effect on SOFA score, the change in SOFA score at time 48 hour post treatment will be used to guide the preferential randomized allocation of subjects to the doses that are the most promising. This approach allows the initial consideration of a wide dose range, but avoids the inefficiency of balanced randomization which would continue to allocate patients to non-promising doses even late in the trial. Because our overall goal is to develop a treatment that decreases the 28-day mortality associated with septic shock, the criteria for assessing the futility or success of the phase II trial will be based on the 28-day mortality benefit seen with the most-promising L-carnitine dose. Specifically, the trial will utilize frequent interim analyses, with ongoing predictive-probability-based assessments of both futility and success, so the trial may be stopped as soon as either: (1) a dose of L-carnitine has been identified that is sufficiently promising to warrant investigation in a phase III trial; or (2) it is clear that none of the three doses is sufficiently effective to warrant further investigation.

The trial will enroll a maximum of 250 subjects allocated to four treatment arms (control, 6 g, 12 g, 18 g). The adaptive trial will utilize both the change in SOFA score at time 48 hour post treatment and 28 day mortality as efficacy endpoints. The allocation of subjects will be determined only by the observed change in SOFA score; however, interpretation of the trial results will be based on both the change in SOFA score and 28-day mortality. For determining the probability of success in a phase III trial, we assume the phase III trial would utilize standard, frequentist approaches and enroll up to a maximum of 2,000 subjects, with half in a control arm and half receiving the selected dose of L-carnitine. A phase III trial of that size would have a power of 95% to detect a reduction of 28-day mortality from 40% to 32% or a power of 78% to detect a reduction from 40% to 34% with L-carnitine.

A normal dynamic linear model (NDLM) is utilized to improve the efficiency in estimating the dose response across adjacent dose levels. During an initial “burn in” period of 40 patients, subjects are allocated equally among the treatment arms. From that point on, an interim analysis is conducted after every 12 subjects and the randomization proportions among the arms are adjusted so that the probability of assignment to each active treatment arm is proportional to the probability that that arm leads to the greatest improvement in SOFA score. A blocked randomization approach is used to ensure that approximately one-third of subjects are allocated to the control arm throughout the trial. This helps to ensure that the result is resistant to confounding due to secular trends in outcome.

The criteria for stopping the trial at each of the interim analyses are based on Bayesian posterior probability calculations. Specifically, the trial is stopped for **futility** if there is less than a 40% probability that the most promising L-carnitine dose leads to an improvement in SOFA at time 48 hour post treatment. The trial may also be stopped early for **success**, if there is a greater than 90% probability that the most promising dose of L-carnitine improves SOFA at time 48 hour post treatment *and* the probability of success in a subsequent phase III trial is greater than 70%. These criteria for defining futility and success at the interim analyses were selected empirically to yield desirable operating characteristics (see Trial Operating Characteristics section).

To estimate the true relationship between the change in SOFA score and 28-day mortality, SOFA score and mortality data from more than 250 subjects with inclusion criteria identical to the proposed trial were obtained from a multicenter observational study. All patients in the source study were treated according to the Surviving Sepsis Campaign Guidelines for the management of septic shock. The observed odds ratio for 28-day mortality, for each decrease in SOFA of one point at 48 hours,

was 1.35 (95% CI 1.20 – 1.53). We used this relationship to generate data for trial simulations. In order to evaluate the operating characteristics and proposed trial design, we conducted Monte Carlo simulations of the trial while assuming a variety of true dose-response relationships for both change in SOFA score and resulting 28-day mortality. For the purposes of simulation, subject enrollment is assumed to ramp up slowly over the first twelve weeks of the trial, reaching a plateau of 1.5 subjects per week. Under each of the scenarios listed in the **Table 1** (no, mild, and strong treatment effects) we simulated thousands of subjects and their corresponding SOFA data. Each simulated subjects' SOFA result was used to determine his or her predicted 28-day mortality, and then a 28-day mortality outcome was randomly assigned with that probability. All trial design work and simulations were completed using the Fixed and Adaptive Clinical Trial Software (FACTS) from Berry Consultants, LLC.

Table 1 shows the resulting trial performance. Under the null hypothesis that there is no relationship between L-carnitine dose and either SOFA score at 48 hours post treatment or 28-day mortality, the trial has a type I error rate of 0.043 and requires an average sample size of 198.0 subjects. The trial was powered to detect an improvement in SOFA of two units at 48 hours post treatment with the highest dose (see column entitled "Mild Treatment Effect"). Under this scenario, the proposed trial has a power of 91.1%

Table 1. Operating Characteristics of Proposed Trial Design: Results of Monte Carlo Simulations (30,000 simulated trials)

	**No Treatment Effect (Null)**		**Mild Treatment Effect**		**Strong Treatment Effect**	
Assumed Treatment Effects for Simulations						
	ΔSOFA	Mortality	ΔSOFA	Mortality	ΔSOFA	Mortality
Outcome: Control	0	40%	0	40%	0	40%
Outcome: 6 g	0	40%	0	40%	-1	34%
Outcome: 12 g	0	40%	-1	34%	-2	28%
Outcome: 18 g	0	40%	-2	28%	-4	19%
Trial Performance						
Probability of Positive Trial	0.043 (type I error rate)		0.911 (power)		0.999	
Probability of [illegible]	For futility: 0.431<br>[illegible] success: 0.023		For futility: 0.001<br>For success: 0.679		For futility: 0.000<br>For success: 0.981	
[illegible]	198.0		172.4		119.5	
[illegible]	0.35		0.99		1.00	
[illegible]ation of Subjects Between Treatment Arms – n per arm (%)						
[illegible]	62.7 (32%)		54.1 (31%)		36.5 (31%)	
[illegible]	47.0 (24%)		13.8 (8%)		10.5 (9%)	
[illegible]	38.7 (20%)		21.5 (12%)		12.5 (10%)	
[illegible]	49.6 (25%)		83.0 (48%)		60.0 (50%)	

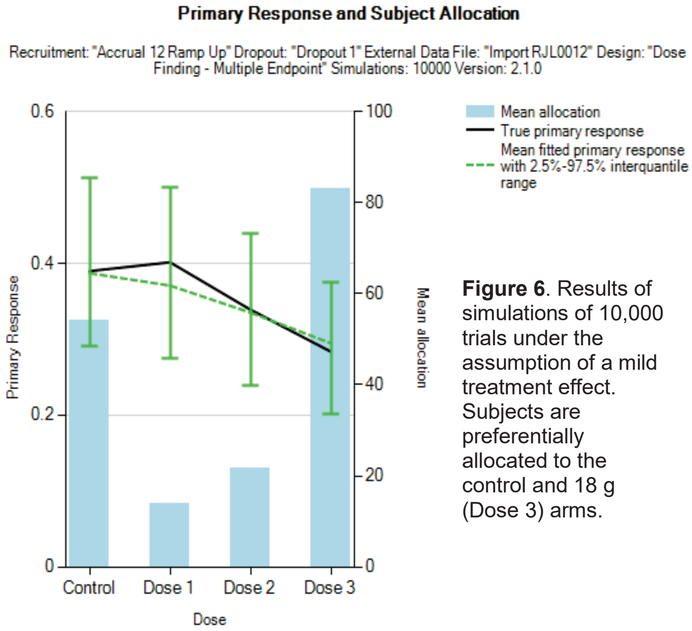

Figure 6. Results of simulations of 10,000 trials under the assumption of a mild treatment effect. Subjects are preferentially allocated to the control and 18 g (Dose 3) arms.

and stops early declaring success over two-thirds of the time. The average required sample size is 172.4 subjects and the design is highly reliable in selecting the highest dose to be carried forward. Moreover, it can be seen that, under this scenario, the trial efficiently allocates to patients to both the control and highest dose arms, maximizing the information gained per subject (**Figure 6**). Table 1 also demonstrates the performance if there is a very strong treatment effect, in which case the power of the trial is even higher and the required sample size smaller.

At the end of the trial, the trial is considered negative if either there is less than a 90% probability that the most promising dose of L-carnitine improves SOFA at 48 hours post treatment or less than a 30% probability that the most promising dose of L-carnitine would be successful in a subsequent phase III trial. The trial is defined as positive if there is a greater than 90% chance that the selected dose of L-carnitine improves SOFA at 48 hours post treatment and a greater than 30% chance of success in a subsequent phase III trial. These criteria for defining futility and success at the interim analyses were selected empirically to yield desirable operating characteristics.

6.0 TRIAL OVERSIGHT

Data Storage and Confidentiality

Data management will be provided by the Biomedical Informatics Group at the University of Mississippi Medical Center. Biomedical Informatics is currently staffed with 5 full time employees, including an executive director (PhD Epidemiology, IT director), database developer (BS Computer Science), data architect (MS Computer Science), application analyst (MS Geosciences), and office manager. Additional positions currently being recruited included manager – IT operations, database administrator, programmer, and data analyst. As a data coordinating center, the Biomedical Informatics group's primary responsibilities are:

- Collaboration with the Center for Biostatistics and Berry Consultants as required for the adaptive trial design to communicate blinded interim data for updating centralized randomization schedule and DSMB reports;
- Staffing for data project management, database administration and data entry training;
- Electronic case report form design and data entry resources;
- Secure online data entry creation and maintenance;
- Design, implementation and maintenance of centralized, web-based, randomization;

- Database design, development, and maintenance, as well as secure data management systems;
- Development and implementation of data quality control procedures;
- Assist in the development of study materials such as data entry SOPs, case report form guidance documents;
- Development and implementation of study-wide systems for communication, coordination, and administration of study activities;
- Data queries and custom report generation.

The Biomedical Informatics Group is currently involved in designing, building, and implementing an enterprise data warehouse for research and quality to provide infrastructure to support research informatics. Epic will be deployed as the electronic medical record at UMMC hospitals and clinics on April 1, 2012. Epic will be a major source of data for the enterprise data warehouse (EDW). The EDW will also house data from approximately thirty legacy systems that will be replaced by Epic and sunsetted. Research databases and datasets will also reside on the EDW. The EDW will be governed by an executive governance committee and several focused working groups.

The Biomedical Informatics group has a close working relationship with the Center for Biostatistics. All data management services, including reports to the Center for Biostatistics for safety monitoring, will be produced with assurance of shielding the clinical management arm from access to unblinded data during performance of the trial. The data management center will also produce performance reports of the sites, which will be communicated to the clinical management center and clinical trial monitor. At the end of the trial, the data management center will clean the data and produce a final locked data set for the PI and participate in the final data analysis and manuscript development.

The data management center will inevitably also handle sensitive data, including information used to monitor the performance and enrollment at each site, and other data as dictated by the needs of the network. Thus the following security procedures will be used.

Standard processes are established to track the flow and quality control of case report forms and data. Data collection will be directly into the electronic database from source documents at the clinical sites. Data entry will be done via REDCap. Quality control will be done during and

post-entry. Discrepancies identified through data review processes will result in query generation sent to clinical sites for resolution. Manual data review may also result in manual queries. Any query will be sent to sites via a data change form (DCF) signed by PI and Coordinator. Changes will then be made to REDCap. The DCF will then be signed-off by the site PI after completion.

Additionally, the Biomedical Informatics group will develop the data management plan to include (but not limited to) the following:

- General CRF completion rules;
- Data entry and processing plans;
- QC - completeness of dates / data ranges for numeric variables / data logic / self-evident corrections;
- Data receipt procedures;
- Conduct web training to sites for CRF completion and provide CRF completion guidelines.

All data are managed on four virtual Linux servers. These servers are managed by a full time database administrator, and are user-id and password protected. These machines are members of a domain server system that are also user id and password protected. Equipment is housed in a building with a security alarm system, which is also patrolled at night by security guards. The data servers and domain servers are behind a firewall that protects them from outside tampering via the Internet. Data housed on the servers are backed up nightly by the administrator of the domain servers. All personnel have permanent office space with desktop computers, laptops, locking file cabinets, and other standard office equipment. All data stored on laptops are encrypted and are in compliance with HIPAA regulations.

In addition to the above facilities, the Biomedical Informatics group has a long-term agreement with the Division of Information Systems (DIS) to provide additional server infrastructure to that provided by the hospital system. The DIS infrastructure provides connectivity with around the clock monitoring, notification and reporting. It utilizes multiple Tier One Internet carriers with redundant fully-lit OCx connections that provide over 1.5 Gbps of bandwidth. Servers are protected by anti-virus software, which is updated automatically on a daily basis with the latest available signature updates. A full system virus scan is automatically performed on a weekly

basis. ProtectPoint Managed Security Services provide intrusion detection and prevention service with integrated managed firewall protection. The Linux-based firewall provides 24x7x365 management, updating, and monitoring and provides Network Address Translations (NAT) to hide internal IP addresses.

The database, system and application files are backed up on a daily incremental basis with full weekly backups. Backup files are stored on site on SANs. Storage areas have controlled temperature and humidity so that the backups are not damaged. The application provides three levels of security – facility, login and password.

No known security breaches have occurred. Written Standard Operating Procedures (SOPs) regarding corrective action plans are maintained. Institutional policies also provide guidance for appropriate corrective action. In the unlikely event of a security breach, a formal written report will be presented to the Study Director and the IRB within 7 days of discovery. The data management center project manager will then work with the Study Director, the Steering Committee, and the IRB to implement an appropriate corrective plan.

Site Monitoring and Quality Assurance

The protection of human subjects is of paramount importance, and the Coordinating Center (CC) will coordinate the human subjects protection plan, data safety, and monitoring the project.

An individual from the CC who is qualified to perform the role of monitor will inspect records of the study periodically. The study monitor will be given access to all source records and data needed to verify the entries on the case report forms (CRFs) to original entries in source files. In addition to the original medical record, the study will employ data collection templates as a source document. These forms contain the data fields that historically have been difficult to abstract from the medical record. Only site personnel designated on a “Delegation of Authority Log” will be authorized to enter data onto the CRF. The investigators agree to cooperate with the study monitor to ensure that any problems detected in the course of monitoring visits are resolved. The monitor will inspect the CRF entries on a predetermined percentage (up to 30%) of subject records to assess the completeness, correctness and accuracy of CRF entries and

to verify adherence to the protocol and applicable regulations. The project research coordinators at the sites will maintain binders containing pertinent documents and information including IRB approval letters, documentation of GCP training, licenses, human subjects research ethics training certificates, curriculum vitae, and correspondence.

Sites will be monitored to ensure their compliance with local IRB approvals and reporting requirements, both prior to initial certification and as part of ongoing performance assessment and quality improvement. All operations will be immediately suspended at any site where ongoing review or approval lapses.

Adverse events will be reported to the CC. The CC will disseminate the information back to both sites for reporting to local IRBs, the sponsor, FDA when necessary, and the data safety monitoring board. This centralized reporting will ensure that unscheduled meetings of the DSMB are called when needed, that there is adherence to previously defined stopping rules, that events are handled in a consistent and timely manner, and that centralized records are available for review at all times.

All serious, study drug-related adverse events will be reported to the CC. SAEs will be reported to the CC by online SAE reporting form that when completed will generate an email to the project manager and Study Director at UMMC. Fatal or life threatening events categorized as "probably related" will be immediately followed up by the Study Director by telephone call to the study site submitting the SAE. The Study Director will notify the FDA within 7 days of learning of the event.

The study requires surveillance for SAEs on a daily basis for seven days after enrollment, and then again at hospital discharge. To determine SAEs at hospital discharge we will record all non-exempt (see below) SAEs that are documented in the hospital discharge summary for the index visit. An SAE will be defined in accordance with ICH-GCP guidelines (any unexpected illness or injury that poses a threat to life or body function and causes medical or surgical intervention or prolongation of hospitalization). Electronic forms will record an estimate of the relationship to the treatment as assessed by the site PI. The study associate will require an email reply from the project manager at the CC that indicates receipt of the SAE. The project manager will disseminate the SAE to both study sites.

DSMB Plan

The data safety monitoring board (DSMB) will follow a charter approved by the sponsor. The DSMB will follow the adaptive rules as outlined in the application unless there is a compelling, unanticipated reason to deviate from these rules. The DSMB may recommend stopping the trial early for one of three reasons, following these guidelines:

[Trial Futility] Stop the accrual of subjects because there is less than a 40% probability that the most promising L-carnitine dose leads to an improvement in SOFA at time 48 hour post treatment.

[Trial Efficacy] Stop the accrual of new subjects because there is a greater than 90% probability that the most promising dose of L-carnitine improves SOFA at time 48 hour post treatment *and* the probability of success in a subsequent phase III trial is greater than 70%.

[Patient Safety] A recommendation to suspend or stop for harm would occur if an interim analysis showed strong evidence that the rate of IRB-reported SAEs was significantly higher in the experimental group than in the placebo group. Because the underlying rate of SAEs will be difficult to predict, it is not possible to provide a table of critical values at each interim meeting that could be used for stopping criteria.

The DSMB will operate in accordance with guidelines established by the FDA in “Guidance for Clinical Trial Sponsors: Establishment and Operation of Clinical Trial Data Monitoring Committee” jointly published by the CBER, CDER and CDRH of the FDA, OMB#0910-0581. The key elements include:

1. Expertise and independence: The members must have expertise in the area of study, not be study authors and have no financial conflict of interest.
2. Members will sign a confidentiality agreement.
3. It is preferred that the DSMB have a face-to-face initial meeting, but can meet by telephone thereafter more frequently.

4. All meetings must follow standard operating procedures (SOPs).

Minutes of all meetings will be filed with the Office of Sponsored Studies at the CC and a copy sent to the other relevant site administrative offices and NIH.

If the DSMB recommends closure of the study for any of the reasons above, then the DSMB Chair will email the Study Director and officials at NIH. Officials at NIH would then decide the action to be taken on the trial.

If the trial is stopped for patient safety and a repairable cause of unacceptably high adverse events can be identified, the action must be reviewed and approved by the DSMB and by each site IRB prior to resuming enrollment. For example, if one additional exclusion criterion is discovered that would account for the majority of unexpected events, this new exclusion criterion could be added, and the study could proceed.

Miscellaneous

Every attempt will be made to report adverse events using the most current version of MedDRA. Subjects who withdraw from study therapy early will be followed in the same manner as other subjects in the study for safety (AEs and SAEs). In that case, other study procedures will not be discontinued unless instructed by the study participant/ LAR.

7.0 REFERENCES

(1) Angus D, Linde-Zwirble W, Lidicker J, Clermont G, Carcillo J, Pinsky M. Epidemiology of severe sepsis in the United States: analysis of incidence, outcome, and associated costs of care. *Crit Care Med* 2001;29:1303-1310.

(2) Centers for Disease Control and Prevention. Cases of HIV infecton and AIDS in the United States, by race/ethnicity, 1998-2002. HIV/AIDS Surveillance Supplemental Report. Centers for Disease Control and Prevention, editor. 10 (1), 5-38. 2003. PO Box 6003 Rockville MD 20849-6003, CDC National Prevention Information Network.

(3) American Cancer Society. Cancer facts and figures 2003. 2003. 1599 Clifton Road NE, Atlanta GA 30329-4251, American Cancer Society Inc.

(4) American Heart Association. Heart disease and stroke statistics - 2004 update. 2004. Dallas TX, American Heart Association.

(5) Kochanek K, Smith B. National Vital Statistics Report. Deaths: Preliminary Data for 2002. Centers for Disease Control and Prevention, editor. Vol 52, No 13, 1-32. 2-11-2004. 3311 Toledo Road Hyattsville MD, US Dept of Health & Human Services. National Vital Statistics Report.

(6) Rosamond W, Folsom A, Chambless L et al. Stroke incidence and survival among middle-aged adults: 9-year follow-up of the Atherosclerosis Risk in Communities (ARIC) cohort. *Stroke* 1999;30:736-743.

(7) Lee K, Woodlief L, Topol E et al. Predictors of 30-day mortality in the era of reperfusion for acute myocardial infarction. Results from an international trial of 41,021 patients. GUSTO-I Investigators. *Circulation* 1995;91:1659-1668.

(8) Goldhaber S, Visani L, De Rosa M. Acute pulmonary embolism: clinical outcomes in the International Cooperative Pulmonary Embolism Registry (ICOPER). *Lancet* 1999;353:1386-1389.

(9) Dombrovskiy VY, Martin AA, Sunderram J, Paz HL. Rapid increase in hospitalization and mortality rates for severe sepsis in the United States: A trend analysis from 1993 to 2003. *Crit Care Med* 2007;35:1244-1250.

(10) Spitzer JJ, Bechtel AA, Archer LT, Black MR, Hinshaw LB. Myocardial substrate utilization in dogs following endotoxin administration. *American Journal of Physiology* 1974;227:132-136.

(11) Dhainaut JF, Huyghebaert MF, Monsallier JF et al. Coronary hemodynamics and myocardial metabolism of lactate, free fatty acids, glucose, and ketones in patients with septic shock. *Circulation* 1987;75:533-541.

(12) Calvani M, Reda E, rrigoni-Martelli E. Regulation by carnitine of myocardial fatty acid and carbohydrate metabolism under normal and pathological conditions. [Review] [78 refs]. *Basic Research in Cardiology* 2000;95:75-83.

(13) Vary TC, Siegel JH, Nakatani T, Sato T, Aoyama H. Effect of sepsis on activity of pyruvate dehydrogenase complex in skeletal muscle and liver. *Am J Physiol* 1986;250:E634-E640.

(14) Ferrannini E, Buzzigoli G, Bevilacqua S et al. Interaction of carnitine with insulin-stimulated glucose metabolism in humans. *American Journal of Physiology* 1988;255:E946-E952.

(15) Biolo G, Toigo G, Ciocchi B et al. Metabolic response to injury and sepsis: changes in protein metabolism. *Nutrition* 1997;13:52S-57S.

(16) Aghajani E, Korvald C, Nordhaug D, Sager G, Revhaug A, Myrmel T. E. Coli sepsis induces profound mechanoenergetic inefficiency in the porcine left ventricle. *Shock* 2004;21:103-109.

(17) Aghajani E, Nordhaug D, Korvald C et al. Mechanoenergetic inefficiency in the septic left ventricle is due to enhanced oxygen requirements for excitation-contraction coupling. *Cardiovascular Research* 2004;63:256-263.

(18) Fukumoto K, Pierro A, Spitz L, Eaton S. Differential effects of neonatal endotoxemia on heart and kidney carnitine palmitoyl transferase I. *J Pediatr Surg* 2002;37:723-726.

(19) Eaton S, Fukumoto K, Stefanutti G, Spitz L, Zammit VA, Pierro A. Myocardial carnitine palmitoyltrasferase 1 as a traget for oxidative modification in inflammation and sepsis. *Biochemical Society Transactions* 2003;31:1133-1136.

(20) Fukumoto K, Pierro A, Zammit VA, Spitz L, Eaton S. Tyrosine nitration of carnitine palmitoyl transferase I during endotoxaemia in suckling rats. *Biochimica et Biophysica Acta* 2004;1683:1-6.

(21) Takeyama N, Takagi D, Matsuo N, Kitazawa Y, Tanaka T. Altered hepatic fatty acid metabolism in endotoxicosis: effect of L-carnitine on survival. *Am J Physiol* 2009;256:E31-E38.

(22) Vary TC. Sepsis-induced alterations in pyruvate dehydrogenase complex activity in rat skeletal muscle: effects on plasma lactate. [abstract]Vary TC. *Shock* 1996;6:89-94

(23) Watts J, Kline J. Bench to bedside: the role of mitochondrial medicine in the pathogenesis and treatment of cellular injury. *Acad Emerg Med* 2003;10:985-997.

(24) Lanza-Jacoby S, Holahan M, Beibel DK. Changes in tissue levels of carnitine during E-coli sepsis in the rat. *Circ Shock* 1988;24:29-34.

(25) Hulsmann WC. Vulnerability of vascular endothelium in lipopolysaccharide toxicity: effect of (acyl) carnitine on endothelial stability. *Mediators Inflamm* 1993;2:S21-S23.

(26) Famularo G, De Simone E, Martelli EA, Jirillo E. Carnitine and septic shock: a review. *J Endotoxin Research* 1995;2:141-147.

(27) Rebouche CJ. Kinetics, pharmacokinetics, and regulation of L-carnitine and acetyl-L-carnitine metabolism. *Ann NY Acad Sci* 2004;1033:30-41.

(28) Nani G, Pittiruti M, Giovannini I, Boldrini G, Ronconi P, Castagneto M. Plasma carnitine levels and urinary carnitine excretion during sepsis. *J Parenter Enteral Nutr* 1985;9:483-490.

(29) Davis AT, Crady SK, Strong SA, Albrecht RM, Scholten DJ. Increased acylcarnitine clearance and excretion in septic rats. *Biomed Biochim Acta* 1991;50:81-86.

(30) Penn D, Zhang L, Bobrowski PJ, Quinn M, Liu X, McDonough KH. Carnitine deprivation adversely affects cardiovascular response to bacterial endotoxin (LPS) in the anesthetized neonatal pig. *Shock* 1998;10:377-382.

(31) Penn D, Zhang L, Bobrowski PJ, Quinn M, McDonough KH. Carnitine deprivation adversley affects cardiac performance in the lipopolysaccharide- and hypoxia reoxygenation-stressed piglet heart. *Shock* 1999;11:120-126.

(32) Yamakawa M, Maeda J, Nakamura T et al. Distribution of endogenous and exogenous carnitine in rats with sepsis and acute liver failure. *Clin Nutr* 1996;15:133-140.

(33) Gallo LL, Tian Y, Orfalian Z, Fiskum G. Amelioration of popolysaccharide-induced sepsis in rats by free and esterified carnitine. *Mediators Inflamm* 1993;2:S51-S56.

(34) Broderick TL, Quinney HA, Lopaschuk GD. L-carnitine increases glucose metabolism and mechanical function folowing ischemia in diabetic rat heart. *Cardiovasc Res* 1995;29:373-378.

(35) Broderick TL, DiDomenico D, Gamble J et al. L-carnitine improvement of cardiac function is associated with a stimulation in glucose but not fatty acid metabolism in carnitine-deficient hearts. *Cardiovasc Res* 1995;30:815-820.

(36) Schonekess BO, Allard MF, Lopaschuk GD. Propionyl L-carnitine improvement of hypertrophied heart function is accompanied by an increase in carbohydrate oxidation. *Circ Res* 1995;77:726-734.

(37) Hulsmann WC, Dubelaar ML. Aspects of fatty acid metabolism in vascular endothelial cells. *Biochimie* 1988;70:681-686.

(38) Steinberg HO, Tarshoby M, Monestel R et al. Elevated circulating free fatty acid levels impair endothelium-dependent vasodilation. *J Clin Invest* 1997;100:1230-1239.

(39) deKreutzenberg SV, Crepaldi C, Marchetto S et al. Plasma free fatty acids and endothelium-dependent vasodilation: effect of chain-length and cyclooxygenase inhibition. *J Clin Endocrinol Metab* 2000;85:793-798.

(40) Hulsmann WC, Peschechera A, Serafini F, Ferrari LE. Release of ischemia in paced rat Langendorff hearts by supply of L-carnitine: role of endogenous long-chain acylcarnitine. *Mol Cell Biochem* 1996;156:87-91.

(41) Hulsmann WC, Dubelaar ML. Carnitine requirement of vascular endothelial and smooth muscle cells in imminent ischemia. *Mol Cell Biochem* 1992;116:125-129.

(42) De Sotomayor MA, Mingorance C, Rodriguez-Rodriguez R, Marhuenda E, Herrera MD. L-carnitine and its propionate: Improvement of endothelial function in SHR through superoxide dismutase-dependent mechanisms. *Free Radical Research* 2007;41:884-891.

(43) Gasparetto A, Corbucci GG, de Blasi RA et al. Influence of acetyl-l-carnitine infusion on haemodynamic parameters and survival of circulatory-shock patients. *Int J Clin Pharm Res* 1991;11:83-92.

(44) Spronk PE, Zandstra DF, Ince C. Bench-to-bedside review: sepsis is a disease of the microcirculation. *Crit Care (London)* 2004;8:462-468.

(45) Trzeciak S, Dellinger RP, Parrillo JE et al. Early microcirculatory perfusion derangements in patients with severe sepsis and septic shock: relationship to hemodynamics, oxygen transport, and survival. *Ann Emerg Med* 1998;49:88-98.

(46) De BD, Creteur J, Preiser JC, Dubois MJ, Vincent JL. Microvascular blood flow is altered in patients with sepsis. *Am J Resp Crit Care Med* 2002;166:98-104.

(47) Sakr Y, Dubois MJ, De BD, Creteur J, Vincent JL. Persistent microcirculatory alterations are associated with organ failure and death in patients with septic shock. *Crit Care Med* 2004;32:1825-1831.

(48) Bateman RM, Sharpe MD, Ellis CG. Bench-to-bedside review: microvascular dysfunction in sepsis--hemodynamics, oxygen transport, and nitric oxide. *Crit Care (London)* 2003;7:359-373.

(49) Farquhar I, Martin CM, Lam C, Potter R, Ellis CG, Sibbald WJ. Decreased capillary density in vivo in bowel mucosa of rats with normotensive sepsis. *J Surg Res* 1996;61:190-196.

(50) Fries M, Weil MH, Sun S et al. Increases in tissue Pco2 during circulatory shock reflect selective decreases in capillary blood flow. *Crit Care Med* 2006;34:446-452.

(51) Lam C, Tyml K, Martin C, Sibbald W. Microvascular perfusion is impaired in a rat model of normotensive sepsis. *J Clin Invest* 1994;94:2077-2083.

(52) Ellis CG, Bateman RM, Sharpe MD, Sibbald WJ, Gill R. Effect of a maldistribution of microvascular blood flow on capillary O(2) extraction in sepsis. *Am J Physiol Heart Circ Physiol* 2002;282:H156-H164.

(53) Groner W, Winkelman JW, Harris AG et al. Orthogonal polarization spectral imaging: a new method for study of the microcirculation. *Nat Med* 1999;5:1209-1212.

(54) Harris AG, Sinitsina I, Messmer K. The Cytoscan Model E-II, a new reflectance microscope for intravital microscopy: comparison with the standard fluorescence method. *J Vasc Res* 2000;37:469-476.

(55) Mathura KR, Vollebregt KC, Boer K, De Graaff JC, Ubbink DT, Ince C. Comparison of OPS imaging and conventional capillary microscopy to study the human microcirculation. *J Appl Physiol* 2001;91:74-78.

(56) Marik PE. Sublingual capnography: a clinical validation study. *Chest* 2001;120:923-927.

(57) Nakagawa Y, Weil MH, Tang W et al. Sublingual capnometry for diagnosis and quantitation of circulatory shock. *Am J Respir Crit Care Med* 1998;157:1838-1843.

(58) Weil MH, Nakagawa Y, Tang W et al. Sublingual capnometry: a new noninvasive measurement for diagnosis and quantitation of severity of circulatory shock. *Crit Care Med* 1999;27:1225-1229.

(59) Creteur J, De Backer D., Sakr Y, Koch M, Vincent JL. Sublingual capnometry tracks microcirculatory changes in septic patients. *Intensive Care Med* 2006;32:516-523.

(60) Jin X, Weil MH, Sun S, Tang W, Bisera J, Mason EJ. Decreases in organ blood flows associated with increases in sublingual PCO2 during hemorrhagic shock. *J Appl Physiol* 1998;85:2360-2364.

(61) Povoas HP, Weil MH, Tang W, Moran B, Kamohara T, Bisera J. Comparisons between sublingual and gastric tonometry during hemorrhagic shock. *Chest* 2000;118:1127-1132.

(62) Povoas HP, Weil MH, Tang W, Sun S, Kamohara T, Bisera J. Decreases in mesenteric blood flow associated with increases in sublingual PCO2 during hemorrhagic shock. *Shock* 2001;15:398-402.

(63) Guzman JA, Lacoma FJ, Kruse JA. Relationship between systemic oxygen supply dependency and gastric intramucosal PCO2 during progressive hemorrhage. *J Trauma Injury Infect Crit Care* 1998;44:696-700.

(64) Shapiro N, Howell MD, Bates DW, Angus DC, Ngo L, Talmor D. The association of sepsis syndrome and organ dysfunction with mortality in emergency department patients with suspected infection. *Ann Emerg Med* 2006;48:583-590.

(65) Levy MM, Macias WL, Vincent JL et al. Early changes in organ function predict eventual survival in severe sepsis. *Crit Care Med* 2005;33:2194-2201.

(66) Vincent JL, Moreno R, Takala J et al. The SOFA (Sepsis-related organ failure assessment) score to describe organ dysfunction/failure. *Intensive Care Med* 1996;*22*:707-710.

(67) Ferreira FL, Bota DP, Bross A, Melot C, Vincent JL. Serial evaluation of the SOFA score to predict outcome in critically ill patients. *JAMA* 2001;286:1754-1758.

(68) Marshall JC, Vincent JL, Guyatt G et al. Outcome measures for clinical research in sepsis: a report of the 2nd Cambridge Colloquium of the International Sepsis Forum. [Review] [73 refs]. *Critical Care Medicine* 2005;33:1708-1716.

(69) Vincent JL. Endpoints in sepsis trials: more than just 28-day mortality?. [Review] [32 refs]. *Critical Care Medicine* 2004;32:S209-S213.

(70) Vincent JL, De BD. Microvascular dysfunction as a cause of organ dysfunction in severe sepsis. [Review] [23 refs]. *Critical care (London, England)* 2005;9:S9-12.

(71) Trzeciak S, Rivers EP. Clinical manifestations of disordered microcirculatory perfusion in severe sepsis. *Crit Care (London)* 2005;9:S20-S26.

(72) Jones AE, Trzeciak S, Kline JA. The Sequential Organ Failure Assessment score for predicting outcome in patients with severe sepsis and evidence of hypoperfusion at the time of emergency department presentation. *Critical Care Medicine* 2009;37:1649-1654.
