# Supplementary material for: Effect of Levocarnitine vs Placebo as an Adjunctive Treatment for Septic Shock: The Rapid Administration of Carnitine in Sepsis (RACE) Randomized Clinical Trial
Source: JAMA Netw Open. 2018 Dec 21;1(8):e186076. doi: 10.1001/jamanetworkopen.2018.6076 (PMC6324339; doi:10.1001/jamanetworkopen.2018.6076)
Supplement: Supplement 2. — Data Sharing Statement [file jamanetwopen-1-e186076-s002.pdf]

Data Sharing Statement

Jones. Effect of Levocarnitine vs Placebo as an Adjunctive Treatment for Septic Shock. *JAMA Netw Open*. Published December 21, 2018. 10.1001/jamanetworkopen.2018.6076

Data
Data available: Yes
Data types: Deidentified participant data
How to access data: Consistent with NIH policy, at the completion of the study award period and all ancillary analyses, data will be de-identified and locked. At that time requests for data sharing can be made to the PI with specific data needs, analysis plans and dissemination plans. Those requests will be reviewed by a study steering committee and the study sponsor for release. Contact: aejones@umc.edu
When available: With publication

Supporting Documents
Document types: Other (please specify)
Additional Information: Trial protocol included with submission documents
How to access documents: Documents provided during submission
When available: With publication

Additional Information
Who can access the data: Requests for data sharing can be made to the PI with specific data needs, analysis plans and dissemination plans. Those requests will be reviewed by a study steering committee and the study sponsor for release.
Types of analyses: For any proposed purpose approved by the study steering committee.
Mechanisms of data availability: After approval of a proposal
Any additional restrictions: All data with be deidentified and protected health information stripped. Dates will be time shifted to eliminate PHI, as needed.
